# Sex differences in neuropsychiatric symptoms in Alzheimer’s disease dementia: a meta-analysis

**DOI:** 10.1186/s13195-022-00991-z

**Published:** 2022-04-04

**Authors:** Willem S. Eikelboom, Michel Pan, Rik Ossenkoppele, Michiel Coesmans, Jennifer R. Gatchel, Zahinoor Ismail, Krista L. Lanctôt, Corinne E. Fischer, Moyra E. Mortby, Esther van den Berg, Janne M. Papma

**Affiliations:** 1 Department of Neurology and Alzheimer Center Erasmus MC, Erasmus MC University Medical Center, PO Box 2040, 3000 CA Rotterdam, The Netherlands; 2 Department of Neurology, Alzheimer Center Amsterdam, Amsterdam University Medical Centers, PO Box 7057, 1007 MB Amsterdam, The Netherlands; 3 Clinical Memory Research Unit, Lund University, Simrisbanvägen 14, 212 24 Malmö, Sweden; 4 Department of Psychiatry, Erasmus MC University Medical Center, PO Box 2040, 3000 CA Rotterdam, The Netherlands; 5 Division of Geriatric Psychiatry, McLean Hospital, Harvard Medical School, 115 Mill St., Belmont, MA 02478 USA; 6 Department of Psychiatry, Massachusetts General Hospital, Harvard Medical School, 25 Shattuck St., Boston, MA 02115 USA; 7 Departments of Psychiatry, Clinical Neurosciences, and Community Health Sciences, Hotchkiss Brain Institute, University of Calgary, 3330 Hospital Dr. NW, Calgary, AB T2N 4N1 Canada; 8 Department of Psychiatry, University of Toronto, Toronto, ON M5S 1A1 Canada; 9 Hurvitz Brain Sciences Research, Sunnybrook Health Sciences Centre, Toronto, ON M4N 3M5 Canada; 10 Keenan Research Centre for Biomedical Science, St. Michael’s Hospital, 36 Queen St. E, Toronto, ON M5B 1W8 Canada; 11 School of Psychology, University of New South Wales, Sydney, NSW 2052 Australia; 12 Neuroscience Research Australia, Sydney, NSW 2031 Australia

**Keywords:** Alzheimer’s disease, Behavioral and psychological symptoms of dementia, Behavioral symptoms, Meta-analysis, Neuropsychiatry, Sex

## Abstract

**Background:**

Neuropsychiatric symptoms (NPS) are common in individuals with Alzheimer’s disease (AD) dementia, but substantial heterogeneity exists in the manifestation of NPS. Sex differences may explain this clinical variability. We aimed to investigate the sex differences in the prevalence and severity of NPS in AD dementia.

**Methods:**

Literature searches were conducted in Embase, MEDLINE/PubMed, Web of Science Core Collection, Cochrane Central Register of Controlled Trials, PsycINFO, and Google Scholar from inception to February 2021. Study selection, data extraction, and quality assessment were conducted in duplicate. Effect sizes were calculated as odds ratios (OR) for NPS prevalence and Hedges’ *g* for NPS severity. Data were pooled using random-effects models. Sources of heterogeneity were examined using meta-regression analyses.

**Results:**

Sixty-two studies were eligible representing 21,554 patients (61.2% females). The majority of the included studies had an overall rating of fair quality (71.0%), with ten studies of good quality (16.1%) and eight studies of poor quality (12.9%). There was no sex difference in the presence of any NPS (*k* = 4, OR = 1.35 [95% confidence interval 0.78, 2.35]) and overall NPS severity (*k* = 13, *g* = 0.04 [− 0.04, 0.12]). Regarding specific symptoms, female sex was associated with more prevalent depressive symptoms (*k* = 20, OR = 1.60 [1.28, 1.98]), psychotic symptoms (general psychosis *k* = 4, OR = 1.62 [1.12, 2.33]; delusions *k* = 12, OR = 1.56 [1.28, 1.89]), and aberrant motor behavior (*k* = 6, OR = 1.47 [1.09, 1.98]). In addition, female sex was related to more severe depressive symptoms (*k* = 16, *g* = 0.24 [0.14, 0.34]), delusions (*k* = 10, *g* = 0.19 [0.04, 0.34]), and aberrant motor behavior (*k* = 9, *g* = 0.17 [0.08, 0.26]), while apathy was more severe among males compared to females (*k* = 11, *g* = − 0.10 [− 0.18, − 0.01]). There was no association between sex and the prevalence and severity of agitation, anxiety, disinhibition, eating behavior, euphoria, hallucinations, irritability, and sleep disturbances. Meta-regression analyses revealed no consistent association between the effect sizes across studies and method of NPS assessment and demographic and clinical characteristics.

**Discussion:**

Female sex was associated with a higher prevalence and greater severity of several specific NPS, while male sex was associated with more severe apathy. While more research is needed into factors underlying these sex differences, our findings may guide tailored treatment approaches of NPS in AD dementia.

**Supplementary Information:**

The online version contains supplementary material available at 10.1186/s13195-022-00991-z.

## Background

Neuropsychiatric symptoms (NPS) are highly prevalent in individuals with Alzheimer’s disease (AD) dementia [1]. Although the majority of individuals with AD dementia exhibit NPS during the course of their disease, there is substantial heterogeneity among individuals regarding the manifestation and evolution of NPS [1, 2].

Emerging research has provided evidence for sex as an important, yet understudied factor that may play an important role in explaining clinical variability within AD dementia [3]. Note that sex refers to the biological and physiological difference between females and males, while gender encompasses the social, environmental, and cultural influences on the biological factors in females and males [4]. Well-known sex differences in AD dementia include the disproportionate higher prevalence and lifetime risk for developing AD dementia in females compared to males [5], with previous studies showing that females are shown to be more vulnerable to AD pathology and AD risk factors compared to males [6–8]. Furthermore, prior research has suggested more severe cognitive deficits and faster cognitive decline among females with AD dementia [8–10].

Prior studies on sex differences in NPS in AD dementia have reported mixed findings. While several studies have suggested that females show a greater and a wider range of NPS [11, 12], others did not to find any sex differences in the prevalence and severity of NPS in AD dementia [13, 14]. When looking at specific NPS, female sex has been related to the presence of affective symptoms and psychotic symptoms [15, 16], whereas apathy and agitation were more prevalent in males [16, 17]. Determining sex differences in NPS prevalence and severity in individuals with AD has important clinical implications [18]. This knowledge may not only aid personalized assessment, but also guide interventions for NPS in AD. Furthermore, sex differences may have health policy and resource allocation implications for NPS screening and management.

To date, sex differences in NPS in AD dementia have not been systematically reviewed. Therefore, we aimed to review the existing literature on sex differences in specific NPS in AD using a meta-analytic approach. In addition, we examined the sources of heterogeneity across studies including study setting, methods of NPS assessment, and demographic and clinical characteristics.

## Methods

This systematic review was preregistered with PROSPERO (CRD42020168064) and conducted conform to the PRISMA guidelines [19].

### Search strategy

In consultation with a research librarian, databases Embase, MEDLINE/PubMed, Web of Science Core Collection, Cochrane Central Register of Controlled Trials, PsycINFO, and Google Scholar were searched from inception to February 2021 (see full search queries in Additional file 1: eTable 1). Studies included in the most recent meta-analysis summarizing the prevalence of NPS in AD dementia were also screened [20]. Reference lists of identified studies were manually checked for potential studies of interest. Finally, experts on the author team were consulted to ensure that no relevant studies were missing.

### Study selection

Articles were screened and selected based on the following criteria: (A) NPS prevalence (dichotomous data) and/or NPS severity (continuous data) for females and males separately. We included papers that referred to both sex differences and gender differences. Furthermore, sex differences had to be reported for either overall NPS burden or specific symptoms and not for clusters of NPS due to its limited comparability. (B) Clinical diagnosis of AD dementia based on either the Diagnostic and Statistical Manual of Mental Disorders (DSM) or International Classification of Diseases (ICD) classification systems or conventional consensus criteria [21, 22]. (C) NPS were assessed using a validated instrument such as the Neuropsychiatric Inventory (NPI) [23] or established using well-defined diagnostic criteria, e.g., depression in AD [24]. (D) Studies had to report sufficient information needed to perform a meta-analysis (e.g., means, standard deviations, frequency tables, and/or odds ratios [OR]). (E) Studies had a cross-sectional observational design. In case of longitudinal data, only baseline data were used. Articles containing small selectively sampled populations were excluded, e.g., sex- and age-matched samples. In cases in which the same cohort of patients was used in different studies, only the study with the largest *N* was selected.

Two independent reviewers (W.S.E., M.P.) screened titles and abstracts, and subsequently inspected full texts for eligibility. Discrepancies were discussed, and consensus was reached (with E.v.d.B.).

### Data extraction

Data of each paper was extracted in duplicate (W.S.E., M.P.). In cases where statistical information was missing, an attempt was made to contact the study’s principal investigator. This was unsuccessful in two studies.

### Quality assessment

Two independent reviewers (W.S.E, M.P.) evaluated the quality of each study using an adjusted quality assessment tool for observational studies from the National Heart, Lung, and Blood Institute (Additional file 1: eTable 2) [25, 26]. Originally, this tool includes 14 quality criteria covering the methodology and study population characteristics. Since we only included cross-sectional studies, we did not evaluate item 7 “Was the time frame sufficient so that one could reasonably expect to see an association between exposure and outcome if it existed?”, item 10 “Was the exposure(s) assessed more than once over time?”, and item 13 “Was loss to follow-up after baseline 20% or less?”. Furthermore, item 14 “Were key potential confounding variables measured and adjusted statistically for their impact on the relationship between exposure(s) and outcome(s)?” was also omitted since studies were not required to include covariates in their analyses.

### Data synthesis and statistical analysis

For this meta-analysis, we studied sex differences in NPS for studies reporting on NPS *prevalence* and NPS *severity*. We examined sex differences in studies that reported the prevalence of any NPS, total scores of NPS measures (e.g., NPI total score), and the prevalence and/or severity for specific NPS analogous to the twelve NPI domains: delusions, hallucinations, agitation/aggression, depressive symptoms, anxiety, euphoria, apathy, disinhibition, irritability, aberrant motor behavior, nighttime behaviors, and eating behaviors [23]. In addition, psychotic symptoms were also studied separately since studies used criteria for psychosis in AD [27], psychosis domain score of the Behavioural Pathology in Alzheimer’s Disease (BEHAVE-AD) Scale [28], or NPI domains of hallucinations and delusions combined [23]. Note that instruments such as the NPI assess *neuropsychiatric symptoms*, while diagnostic criteria such as psychosis in AD or DSM diagnosis of a major depressive episode capture *neuropsychiatric syndromes*. In our analyses, these assessment methods will initially be combined and denoted as symptoms. Next, meta-regression analyses will be used to examine the differences in the outcomes between studies that used questionnaires (symptoms) and studies that used diagnostic criteria (syndromes).

For the studies that reported on NPS prevalence, ORs were calculated based on the 2 × 2 frequency tables based on the following formula: $\mathrm{OR}=\frac{\left({\mathrm{NPS}}_{\mathrm{females}}/\mathrm{non}-{\mathrm{NPS}}_{\mathrm{females}}\right)}{\left({\mathrm{NPS}}_{\mathrm{males}}/\mathrm{non}-{\mathrm{NPS}}_{\mathrm{males}}\right)}$. An OR = 1 represents that there is no sex difference in NPS, whereas an OR > 1 suggests that female sex is associated with higher odds of having NPS and an OR < 1 suggest that male sex is associated with higher odds of having NPS. For the studies that reported on NPS severity, means and standard deviations were converted into Hedges’ *g* using the following formula: *g* = $\frac{M_1-{M}_2}{{\mathrm{SD}}_{\mathrm{pooled}}}$, where SD_pooled_ was calculated based on the following formula: ${\mathrm{SD}}_{\mathrm{pooled}}=\sqrt{\frac{{\mathrm{SD}}_1^2+{\mathrm{SD}}_2^2}{2}}$. If studies did not report the means and standard deviations, reported effect sizes were converted to Hedges’ *g* using conventional formulas [29]. A positive effect size indicates more severe NPS for women compared to men.

Heterogeneity was assessed with the *I*^2^ statistic and tested using Cochran’s *Q*-test [30]. The *I*^2^ statistic is an appraisal of the consistency of the effect sizes: > 25% suggests low, > 50% suggests moderate, and > 75% suggests high inconsistency across studies. In case of a significant *Q* statistic and moderate or high inconsistency across studies, we conducted outliers/influential study diagnostics. Influential studies were identified if one of the following was true: DFFITS value > 3√(*p*/(*k* − *p*)) where *p* is the number of model coefficients and *k* is the number of studies, lower tail of a chi-square distribution of *p* degrees of freedom cutoff by the Cook’s distance > 50%, hat value > 3(*p*/*k*), and/or the DFBETAS value > 1 [31]. In case influential cases were identified, leave-1-out meta-analyses were conducted to examine how individual studies affected the summary statics. Based on these analyses and visual examination of the forest plots, we excluded one study in the meta-analysis for studies reporting on the prevalence of any NPS, one study in the meta-analysis on psychotic symptoms prevalence, one study in the meta-analysis on irritability prevalence, one study in the meta-analysis on agitation prevalence, and one study in the meta-analysis on aberrant motor behavior prevalence (see Additional file 1: eTable 8). For meta-analyses on NPS severity, one study was identified as an outlier in the meta-analyses on the total scores of NPS measures, agitation, aberrant motor behavior, anxiety, apathy, delusions, depressive symptoms, disinhibition, euphoria, and hallucinations (see Additional file 1: eTable 8).

The following meta-regression and subgroup analyses were selected a priori: study setting (community-based vs. clinic sample), clinical relevance (neuropsychiatric symptoms vs. a clinically relevant cutoff score or clinical criteria for NPS syndrome), method of NPS assessment (proxy vs. self-reported), NPI vs. non-NPI measures, mean age of patients, mean years of education of patients, mean Mini-Mental State Examination (MMSE) score, mean disease duration in years, percentage of *APOE*-ε4 carriers, and study quality (poor/fair/good). In addition, we ran subgroup analyses for studies reporting significant sex differences in age, MMSE score, proportion *APOE*-ε4 carriers, and/or disease duration compared to studies that did not find sex differences in these characteristics. We tested whether the heterogeneity across studies was explained by these moderators using omnibus Wald-type tests. We conducted meta-regression analyses including studies that were identified as outliers and only if a minimum of six studies was available for continuous moderators and at least four studies were available for each subgroup of categorical moderators [32].

Funnel plot asymmetry was evaluated as an indication for publication bias. Begg’s rank tests and Egger’s regression tests were used to test for funnel plot asymmetry. If any of these tests was indicative of funnel plot asymmetry, the trim-and-fill method was used to estimate the number of missing studies and to recompute the summary statistics based on complete data [33].

In order to aggregate studies that reported multiple independent outcomes, we used multilevel meta-analyses including a random factor for study. Multilevel meta-analyses were conducted for 18 outcomes across the 17 studies that reported the severity of depressive symptoms. Because substantial heterogeneity between studies was expected, random-effects models were applied for all analyses. All analyses were conducted using the *metafor* package in *R* v4.0 [34].

## Results

### Characteristics of included studies

**Fig. 1:**
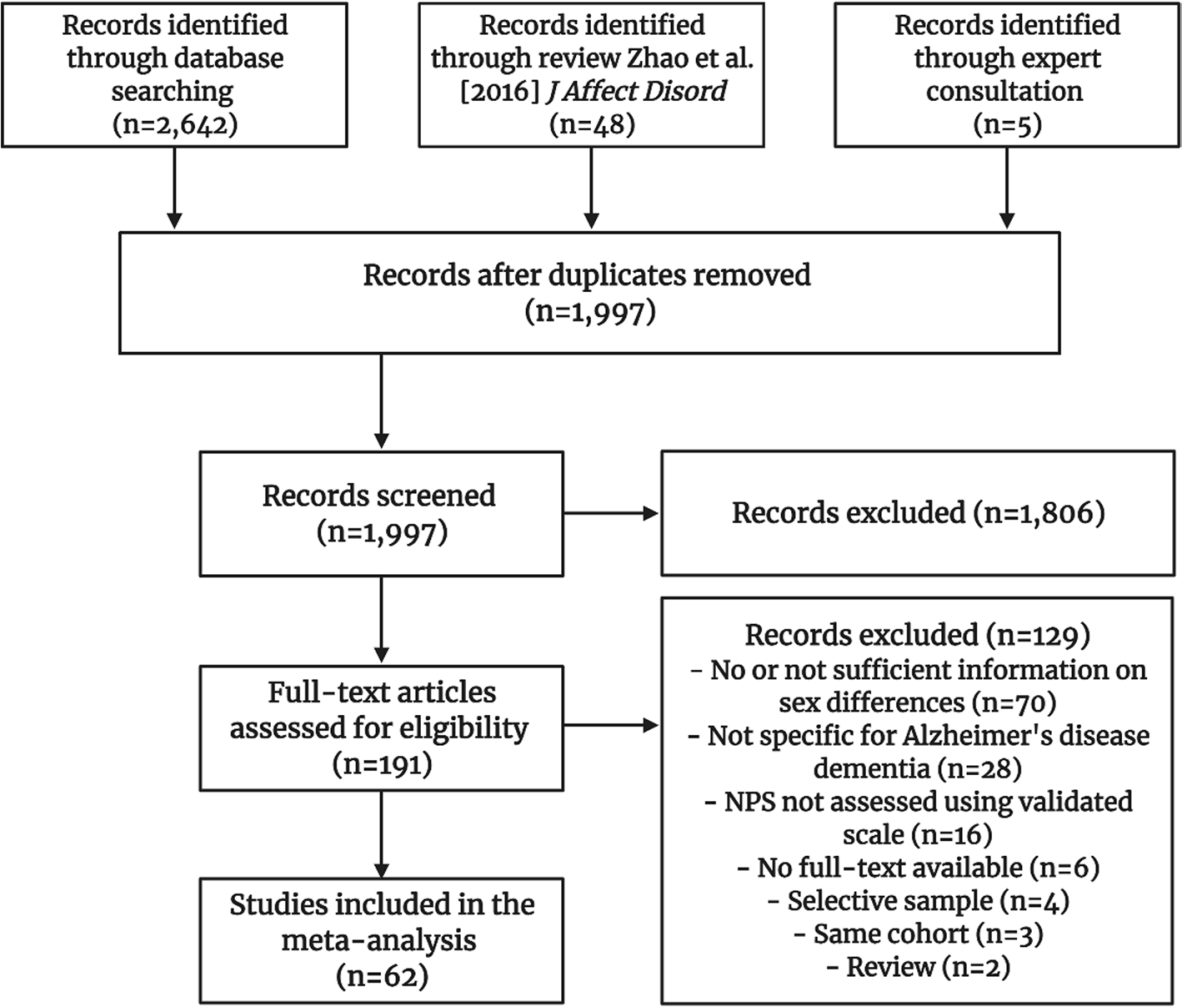
PRISMA flow diagram of the literature search. Note: created with BioRender.com

A total of 1997 unique articles were obtained and screened for eligibility (Fig. 1). Next, the full texts of 191 records were reviewed, of which 62 met all the inclusion criteria (Additional file 1: eTable 3).

The 62 studies included 21,554 individuals with AD dementia, including 13,201 (61%) females and 8353 (39%) males. The majority of studies assessed NPS using a proxy instrument (*k* = 49, 79%), of which 31 used the NPI and four used its questionnaire form. Six studies additionally used self-report scales (10%). In eight studies (13%), clinicians established NPS based on a DSM diagnosis, an ICD-9 diagnosis, or criteria for depression in AD [24], psychosis in AD [27], or apathy in AD [35]. Information on the characteristics of the informant who rated NPS was reported in four studies [36–39], of which two reported these characteristics for male and female patients separately [37, 38]. The majority of the informants were the spouse [36–39], which was primarily the case for male patients (66–86% for male patients and 21–38% in female patients) [37, 38]. The majority of caregivers were female [36–39], although to a lesser extent for female patients (90% for male patients and 61% for female patients) [37]. Clinical AD diagnoses were supported by positive AD biomarkers in subsamples of only two studies. Information on *APOE*-ε4 status was reported in 13 studies, and percentage *APOE*-ε4 carriers ranged from 22% to 68% (Additional file 1: eTable 3). Forty studies provided dichotomous NPS measures, while 17 studies reported continuous NPS measures and five studies reported both dichotomous and continuous outcomes. This resulted in 43 studies reporting on NPS prevalence and 22 studies reporting on NPS severity.

### Study quality

The majority of the included studies had an overall rating of fair quality (44, 71%), with ten studies of good quality (16%) and eight studies of poor quality (13%) (Additional file 1: eTable 2).

### Sex differences in any NPS and total scores of NPS measures

**Fig. 2:**
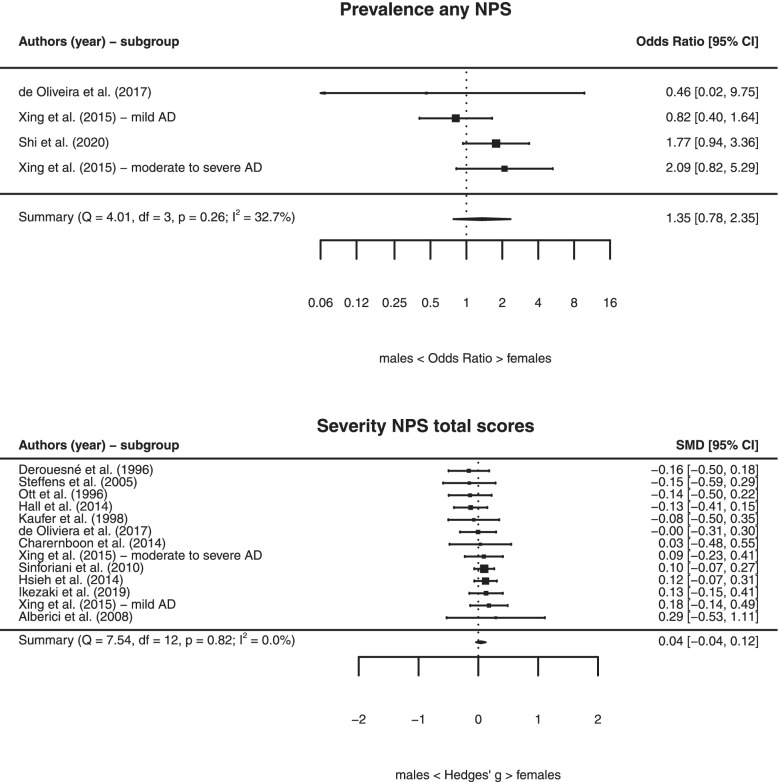
Forest plots for the prevalence of any NPS and severity of NPS total scores. *Abbreviations*: AD, Alzheimer’s disease; NPS, neuropsychiatric symptoms

There was no sex difference in the prevalence of any NPS (*k* = 4, OR = 1.35 [95% CI, 0.78, 2.35], *P* = 0.28), with low heterogeneity across studies (*I*^2^ = 32.74%, *Q* = 4.01, *P* = 0.25) (Table 1 and Fig. 2). We also found no relationship between sex and total severity scores of NPS instruments (*k* = 13, *g* = 0.04 [− 0.04, 0.12], *P* = 0.31), with low heterogeneity across studies (*I*^2^ = 0.00%, *Q* = 7.54, *P* = 0.82) (Table 2 and Fig. 2).

**Table 1:** Sex differences in the prevalence of neuropsychiatric symptoms in Alzheimer’s disease dementia

NPS	***k***	OR [95% CI]^**a**^	***z*** statistic	***P***	***Q*** statistic	***P Q*** statistic	***I***^**2**^ statistic
Any NPS present (outlier excluded)	4	1.35 [0.78, 2.35]	1.07	0.28	4.01	0.25	32.74
Psychotic symptoms (outlier excluded)	4	1.62 [1.12, 2.33]	2.56	0.01	1.98	0.58	0.00
Depressive symptoms	20	1.60 [1.28, 1.98]	4.20	< 0.001	51.99	< 0.001	58.19
Delusions	12	1.56 [1.28, 1.89]	4.45	< 0.001	8.51	0.67	0.00
Aberrant motor behavior (outlier excluded)	6	1.47 [1.09, 1.98]	2.53	0.01	2.51	0.78	0.00
Anxiety	8	1.42 [0.74, 2.71]	1.05	0.29	23.37	0.00	78.49
Eating behavior	5	1.31 [0.97, 1.76]	1.78	0.08	5.40	0.25	22.00
Disinhibition	8	1.17 [0.80, 1.70]	0.81	0.42	13.54	0.06	42.07
Irritability (outlier excluded)	5	1.14 [0.83, 1.56]	0.80	0.43	6.11	0.19	0.00
Hallucinations	9	1.03 [0.79, 1.35]	0.24	0.81	9.89	0.27	14.23
Agitation (outlier excluded)	10	1.00 [0.75, 1.35]	0.01	0.99	16.63	0.06	46.06
Euphoria	6	0.98 [0.57, 1.68]	− 0.08	0.93	6.56	0.26	14.77
Apathy	12	0.92 [0.73, 1.17]	− 0.65	0.51	17.66	0.09	36.92
Sleep disturbances	8	0.86 [0.63, 1.16]	− 0.99	0.32	14.49	0.04	62.49

*Abbreviations*: *k* number of studies, *NPS* neuropsychiatric symptoms

^a^*OR* odds ratio. OR = 1 no sex differences; OR > 1 female sex associated with NPS; OR < 1 male sex associated with NPS

**Table 2:** Sex differences in the severity of neuropsychiatric symptoms in Alzheimer’s disease dementia

NPS	***k***	Hedges’ ***g*** [95% CI]^**a**^	***z*** statistic	***P***	***Q*** statistic	***P Q*** statistic	***I***^**2**^ statistic
Total score NPS measure (outlier excluded)	13	0.04 [− 0.04, 0.12]	1.03	0.31	7.54	0.82	0.00
Depressive symptoms (outlier excluded)	16	0.24 [0.14, 0.34]	4.59	< 0.001	30.15	0.02	44.29
Delusions (outlier excluded)	10	0.19 [0.04, 0.34]	2.53	0.01	19.99	0.02	58.78
Aberrant motor behavior (outlier excluded)	9	0.17 [0.08, 0.26]	3.56	< 0.001	3.25	0.92	0.00
Anxiety (outlier excluded)	10	0.11 [0.00, 0.22]	1.98	0.05	13.27	0.01	25.15
Sleep disturbances	6	0.11 [− 0.02, 0.24]	1.62	0.11	5.66	0.34	21.77
Disinhibition (outlier excluded)	10	0.08 [− 0.05, 0.21]	1.16	0.25	17.01	0.05	46.48
Eating behavior	6	0.07 [− 0.04, 0.18]	1.28	0.20	3.23	0.67	0.00
Hallucinations (outlier excluded)	10	0.07 [− 0.13, 0.26]	0.65	0.51	36.63	< 0.001	77.20
Agitation (outlier excluded)	11	0.01 [− 0.07, 0.10]	0.26	0.79	12.53	0.25	3.12
Irritability	11	0.00 [− 0.08, 0.07]	− 0.10	0.92	14.91	0.14	0.00
Euphoria (outlier excluded)	10	0.00 [− 0.10, 0.10]	− 0.04	0.97	8.10	0.52	14.55
Apathy (outlier excluded)	11	− 0.10 [− 0.18, − 0.01]	− 2.25	0.02	5.00	0.89	0.00

*Abbreviations*: *k* number of studies, *NPS* neuropsychiatric symptoms

^a^Hedges’ *g* = 0 no sex differences; Hedges’ *g* > 0 female sex associated with NPS; Hedges’ *g* < 0 male sex associated with NPS

### Sex differences in the prevalence of specific NPS

**Fig. 3:**
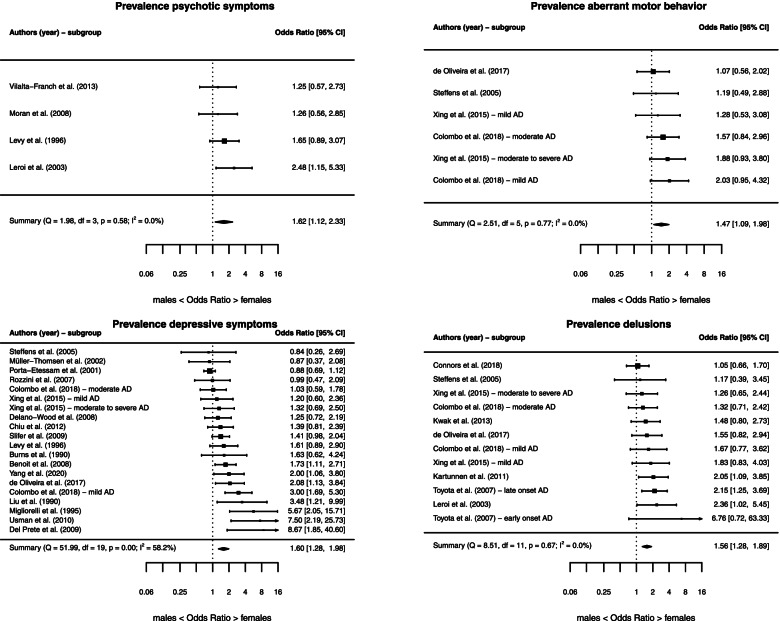
Forest plots for the prevalence of specific neuropsychiatric symptoms. *Abbreviations*: AD, Alzheimer’s disease

We observed a higher prevalence among females compared to males for psychotic symptoms (*k* = 4, OR = 1.62 [1.12, 2.33], *P* = 0.01), depressive symptoms (*k* = 20, OR = 1.60 [1.28, 1.98], *P <* 0.001), delusions (*k* = 12, OR = 1.56 [1.28, 1.89], *P <* 0.001), and aberrant motor behavior (*k* = 6, OR = 1.47 [1.09, 1.98], *P* = 0.01) (Fig. 3). The heterogeneity across the studies included in these meta-analyses was moderate for depressive symptoms (*I*^2^ = 58.19%, *Q* = 51.99, *P* < 0.001), but low for the meta-analyses on psychotic symptoms (*I*^2^ = 0.00%, *Q* = 1.98, *P* = 0.58), delusions (*I*^2^ = 0.00%, *Q* = 8.51, *P* = 0.67), and aberrant motor behavior (*I*^2^ = 0.00%, *Q* = 2.51, *P* = 0.78). There were no significant sex differences in the prevalence of the remaining NPS (Table 1 and Additional file 1: eFigure 1).

### Sex differences in the severity of specific NPS

**Fig. 4:**
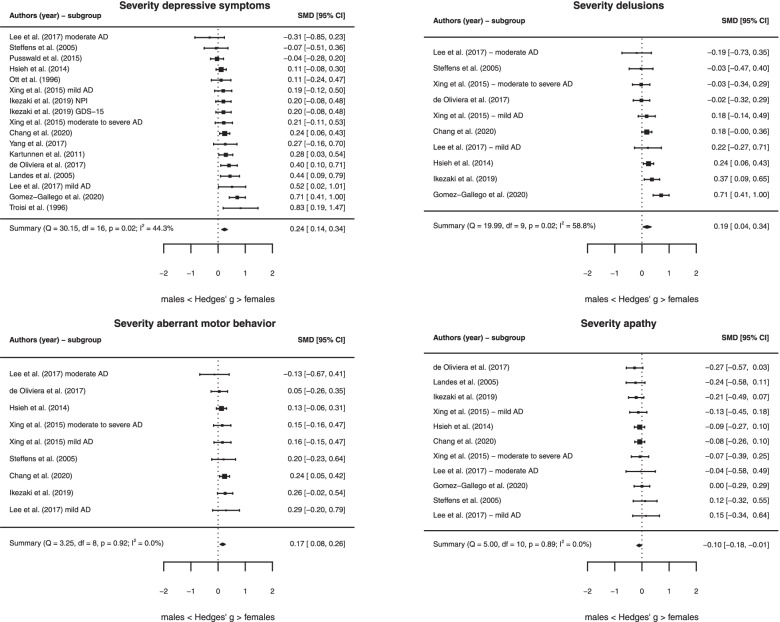
Forest plots for the severity of specific neuropsychiatric symptoms. *Abbreviations*: AD, Alzheimer’s disease

The results showed that female sex was associated with more severe depressive symptoms (*k* = 16, *g* = 0.24 [0.14, 0.34], *P <* 0.001), delusions (*k* = 10, *g* = 0.19 [0.04, 0.43], *P* = 0.01), and aberrant motor behavior (*k* = 9, *g* = 0.17 [0.08, 0.26], *P <* 0.001). Furthermore, apathy was more severe among males compared to females (*k* = 11, *g* = − 0.10 [− 0.18, − 0.01], *P* = 0.02) (Fig. 4). We found moderate heterogeneity across studies including in the meta-analyses on delusions (*I*^2^ = 58.78%, *Q* = 19.99, *P* = 0.02) and depressive symptoms (*I*^2^ = 44.29%, *Q* = 30.15, *P* = 0.02), while heterogeneity was low for aberrant motor behavior (*I*^2^ = 0.00%, *Q* = 3.25, *P* = 0.92) and apathy (*I*^2^ = 0.00%, *Q* = 5.00, *P* = 0.89). There were no significant sex differences in the severity of the remaining NPS (Table 2 and Additional file 1: eFigure 2).

### Meta-regression analyses

We did not find any consistent association between effect sizes across studies and clinical relevance (symptoms vs. syndromes), NPI vs. non-NPI measures, years of education, MMSE score, proportion *APOE*-ε4 carriers, and study quality (poor/fair/good) (Additional file 1: eTable 4 and eTable 5). Meta-regression analysis was not possible for study setting (community vs. clinic-based samples) because there was a paucity of studies with community samples available, and meta-regression for method of NPS assessment (proxy vs. self-report) was only possible for depressive symptoms but showed no difference.

Due to insufficient data, we were not able to compare the effect sizes on NPS prevalence of studies reporting significant sex differences in demographic or clinical characteristics with studies that did not. For all studies combined reporting on NPS severity, we found comparable effect sizes when comparing studies that reported significantly lower MMSE scores for females compared to males (*k* = 5, *g* = 0.39 [− 0.19, 0.97]) with studies that reported no sex differences in MMSE scores (*k* = 10, *g* = 0.38 [− 0.14, 0.89], *QM* = 0.00, *P* = 0.97). Of the 20 studies that tested the sex differences in age, only two reported older age among females and one study reported younger age in females compared to males. Nine studies tested the sex differences in *APOE* status, and three found a higher percentage of *APOE*-ε4 carriers among females. All five studies that compared disease duration between females and males found no sex difference.

### Publication bias

**Fig. 5:**
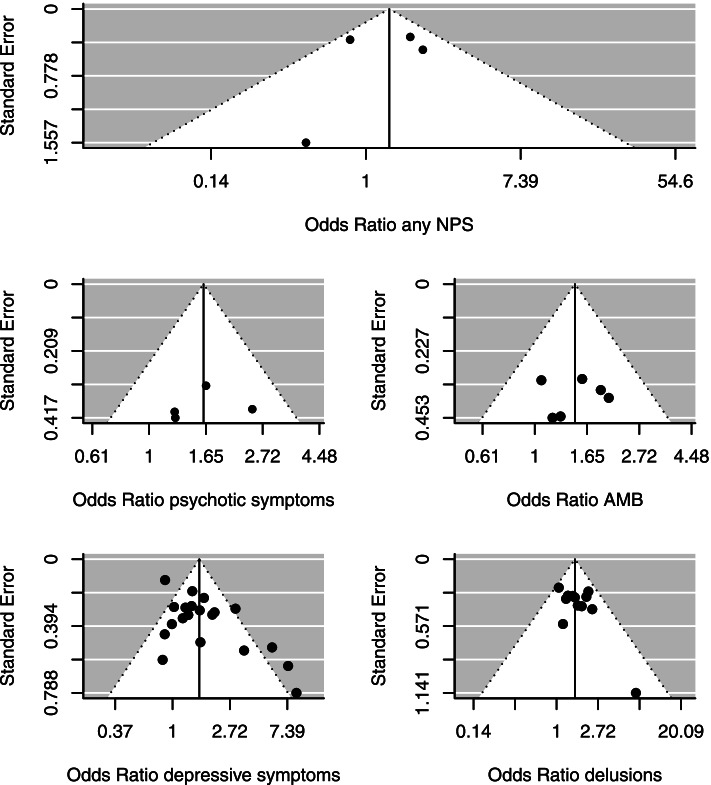
Funnel plots for the prevalence of neuropsychiatric symptoms. *Abbreviations*: AMB, aberrant motor behavior; NPS, neuropsychiatric symptoms

**Fig. 6:**
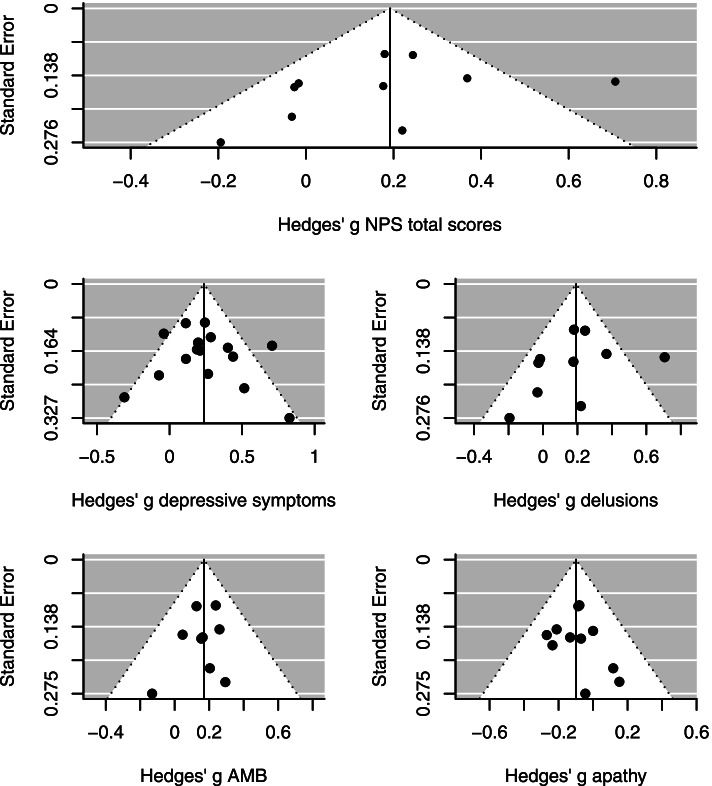
Funnel plots for the severity of neuropsychiatric symptoms. *Abbreviations*: AMB, aberrant motor behavior; NPS, neuropsychiatric symptoms

Begg’s rank test and Egger’s regression test indicated funnel plot asymmetry for the meta-analysis on the prevalence of depressive symptoms and the prevalence of agitation (Additional file 1: eTable 6) (Additional file 1: eFigure 3). However, publication bias was considered less likely as similar estimates were obtained when adjusting for potential publication bias using trim-and-fill method (Additional file 1: eTable 7). We found no indication of publication bias for the remaining meta-analyses (Figs. 5 and 6, Additional file 1: eFigure 4).

## Discussion

Our meta-analysis suggests that female sex is associated with a higher prevalence and greater severity of depressive symptoms, aberrant motor behavior, and psychotic symptoms in AD dementia, while male sex is related to increased severity of apathy in AD dementia. These associations were robust and generally not affected by characteristics relating to the study sample or the method of NPS measurement.

With this meta-analysis, we provide further evidence for greater NPS burden in females with AD dementia found in prior studies [11, 12, 15, 16] and increased severity of apathy among males with AD dementia [16]. However, we found no evidence for higher prevalence rates of agitation/aggression in males that have been reported previously [17]. Sex differences in affective symptoms in AD dementia are in line with higher prevalence rates of lifetime anxiety and mood disorders among females in the general population [40]. Studies on sex differences in psychotic symptoms in the general population have generally shown higher prevalence rates among males [41], which is in contrast to the findings of our meta-analysis in AD dementia. The sex differences observed in this meta-analysis may be explained in part by a prior history of psychiatric illness, although we were not able to verify this as the included studies did not report lifetime history of psychiatric illnesses. Yet, emergent psychiatric symptoms are common symptoms in AD [1, 20] and cannot be fully explained by prior psychiatric disorders but are also related to neurobiological and psychosocial factors associated with AD.

Sex differences in genetics and neurodegenerative and pathophysiologic processes related to AD may partly explain the observed associations, as previous studies have indicated greater amyloid-*β* burden, tau pathology, and loss of brain volume in females compared to males [6–8]. In addition, sex differences in *APOE* status may also contribute to the differences found in NPS. However, prior studies have reported inconsistent associations between NPS and AD-related biomarkers and *APOE* ε4 carriership (e.g., [42, 43]), suggesting that neurobiological factors alone cannot explain these sex differences. Several other biological and medical factors including sex hormones and cardiovascular disease have been related to sex differences in the risk for AD dementia and its clinical manifestation (e.g., [44, 45]). Whether and how these factors may play a role in sex differences in NPS in AD dementia warrants further investigation.

Sex differences in NPS may also be explained by the differences in other clinical and demographic characteristics in AD dementia [10, 18]. For example, females may exhibit more NPS as prior studies suggested that females may be diagnosed later in the disease process potentially leading to more symptoms at diagnosis [46]. Included samples in our study did not reveal sex differences in disease duration and we found comparable results when accounting for the sex differences in MMSE. Although a few studies have shown that associations between sex and NPS were independent of characteristics such as age, education level, cognitive functioning, and ethnicity (e.g., [11, 15]), more studies are needed to examine how sex differences in the clinical and demographic characteristics contribute to sex differences in NPS in AD dementia. Moreover, as NPS were most often assessed using proxy instruments, it would also be interesting to compare informant characteristics for female and male patients. However, only two of the 62 included studies reported these characteristics for female and male patients separately making it impossible to examine whether informant characteristics affected our findings.

The findings of this study may have important implications. First, our findings suggest that sex is a differential factor explaining interindividual differences in the prevalence and severity of specific NPS. These findings may guide the early detection of specific NPS in AD dementia. Second, our results may provide a starting point in informing underlying mechanisms of NPS in AD dementia. More research is needed to study why females with AD are more prone to exhibit significant depressive symptoms, aberrant motor behavior, and psychotic symptoms, and why males are more prone to display severe apathy. Potentially, this research may provide insight into the sex-related differences in neurobiological mechanisms, medical conditions, and cultural factors including gender roles underlying the interindividual differences in the manifestation of NPS in AD dementia. In addition, both pharmacological and psychosocial treatment approaches for NPS in AD dementia are currently identical for females and males. Determining if the sex differences we observed in NPS are subserved by different underlying neurobiological and/or psychosocial mechanisms is critical to personalize treatment. If differences do exist, they could inform sex-specific pharmacological and non-pharmacological intervention that target NPS in AD dementia [47, 48].

This study has some limitations. First, we used meta-regression analyses in order to investigate sources of heterogeneity across studies. Although this approach is commonly used, meta-regression analyses should be interpreted with caution as these analyses may have low power and are prone to ecological bias, i.e., a relationship found at the sample level may not represent the individual level [49]. Second, in case of substantial heterogeneity across studies, we decided to exclude outliers or otherwise influential studies, i.e., based on low number of participants or disproportionate males to females ratio (Additional file 1: eTable 8 and eTable 9) [50]. Although most researchers emphasize the importance of examining the potential outliers and influential studies when confronted with substantial heterogeneity across studies, outlier diagnostics remain under debate in the context of meta-analyses [30]. Third, the majority of the included samples were derived from memory clinics and day care centers, while nursing home populations were not available. Fourth, only two studies supported AD dementia diagnoses with AD biomarkers, whereas the remaining studies used solely a clinical diagnosis of AD dementia and thereby increasing the likelihood of including other etiologies than AD. Finally, the majority of the included studies primarily established NPS based on proxy-based instruments. To further support our findings, future studies are needed in which AD diagnoses are validated by AD biomarkers and the presence of NPS are based on updated diagnostic criteria [51–53]. Finally, it remains unclear whether the associations between sex and NPS in AD dementia change during the course of the disease as we investigated these relationships using cross-sectional data. Future longitudinal studies are needed to provide more insight into the effects of sex on NPS over the course of AD dementia.

## Conclusion

In AD dementia, female sex is associated with greater prevalence and severity of depressive symptoms, psychotic symptoms, and aberrant motor behavior, while males exhibit more severe apathy compared to females. While more research is needed to identify factors underlying the sex differences in NPS in AD dementia, these findings may guide tailored treatment approaches of NPS in AD dementia.

## Supplementary Information

**Additional file 1: eTable 1.** Search strategy literature search. **eTable 2.** Study quality assessment. **eTable 3.** Characteristics of included studies. **eTable 4.** Meta-regression analyses prevalence specific NPS. **eTable 5.** Meta-regression analyses severity specific NPS. **eTable 6.** Publication bias measures for all meta-analyses. **eTable 7.** Duval and Tweedie’s trim-and-fill method to adjust for publication bias. **eTable 8.** Sex differences in the prevalence NPS for meta-analyses that excluded outliers. **eTable 9.** Sex differences in the severity of NPS for meta-analyses that excluded outliers. **eFigure 1.** Forest plots for meta-analyses prevalence specific NPS. **eFigure 2.** Forest plots for meta-analyses severity specific NPS. **eFigure 3.** Funnel plots for meta-analyses prevalence specific NPS. **eFigure 4**. Funnel plots for meta-analyses severity specific NPS.

## Data Availability

The datasets supporting the conclusions of this article are available upon reasonable request.

## Abbreviations

AD: Alzheimer’s disease
BEHAVE-AD: Behavioural pathology in Alzheimer’s disease
DSM: Diagnostic and Statistical Manual of Mental Disorders
ICD: International Classification of Diseases
MMSE: Mini-Mental State Examination
NPI: Neuropsychiatric Inventory
NPS: Neuropsychiatric symptoms
OR: Odds ratio
