# Supplementary material for: Sex differences in neuropsychiatric symptoms in Alzheimer’s disease dementia: a meta-analysis
Source: Alzheimers Res Ther. 2022 Apr 4;14:48. doi: 10.1186/s13195-022-00991-z (PMC8978393; doi:10.1186/s13195-022-00991-z)
Supplement: Supplementary file 1 — Additional file 1: eTable 1. Search strategy literature search. eTable 2. Study quality assessment. eTable 3. Characteristics of included studies. eTable 4. Meta-regression analyses prevalence specific NPS. eTable 5. Meta-regression analyses severity specific NPS. eTable 6. Publication bias measures for all meta-analyses. eTable 7. Duval and Tweedie’s trim-and-fill method to adjust for publication bias. eTable 8. Sex differences in the prevalence NPS for meta-analyses that excluded outliers. eTable 9. Sex differences in the severity of NPS for meta-analyses that excluded outliers. eFigure 1. Forest plots for meta-analyses prevalence specific NPS. eFigure 2. Forest plots for meta-analyses severity specific NPS. eFigure 3. Funnel plots for meta-analyses prevalence specific NPS. eFigure 4. Funnel plots for meta-analyses severity specific NPS. [file 13195_2022_991_MOESM1_ESM.pdf]

Sex differences in neuropsychiatric symptoms in Alzheimer's disease: A meta-analysis

Willem S. Eikelboom[a]*, Michel Pan[a], Rik Ossenkoppele[b,c], Michiel Coesmans[d], Jennifer R. Gatchel[e,f], Zahinoor Ismail[g], Krista L. Lanctôt[h,i], Corinne E. Fischer[j,k], Moyra E. Mortby[l,m], Esther van den Berg[a], Janne M. Papma[a]

[a] *Department of Neurology and Alzheimer center Erasmus MC, Erasmus MC, University Medical Center, PO Box 2040, 3000 CA, Rotterdam, the Netherlands*
[b] *Department of Neurology, Alzheimer Center Amsterdam, Amsterdam University Medical Center, PO Box 7057, 1007 MB, Amsterdam, the Netherlands*
[c] *Clinical Memory Research Unit, Lund University, Simrisbanvägen 14, 212 24, Malmö, Sweden*
[d] *Department of Psychiatry, Erasmus MC, University Medical Center, PO Box 2040, 3000 CA, Rotterdam, the Netherlands*
[e] *Division of Geriatric Psychiatry, McLean Hospital, Harvard Medical School, 115 Mill St, Belmont, MA 02478, Belmont, Massachusetts, USA*
[f] *Department of Psychiatry, Massachusetts General Hospital, Harvard Medical School, 25 Shattuck St, Boston, MA 02115, Boston, Massachusetts, USA*
[g] *Departments of Psychiatry, Clinical Neurosciences, and Community Health Sciences, Hotchkiss Brain Institute, University of Calgary, 3330 Hospital Dr NW, AB T2N 4N1, Calgary, Alberta, Canada*
[h] *Department of Psychiatry, University of Toronto, 27 King's College Cir, ON M5S 1A1, Toronto, Canada*
[i] *Hurvitz Brain Sciences Research, Sunnybrook Health Sciences Centre, Wellness Way, ON M4N 3M5, Toronto, Canada*
[j] *Keenan Research Centre for Biomedical Science, St. Michael's Hospital, 36 Queen St E, ON M5B 1W8, Toronto, Canada*
[k] *School of Psychology, University of New South Wales, Kensington NSW 2052, Australia*
[l] *Neuroscience Research Australia, Sydney, 139 Barker St, Randwick NSW 2031, Australia*

Supplemental materials

eTable 1. **Search strategy literature search**
eTable 2. **Study quality assessment**
eTable 3. **Characteristics of included studies**
eTable 4. **Meta-regression analyses prevalence specific NPS**
eTable 5. **Meta-regression analyses severity specific NPS**
eTable 6. **Publication bias measures for all meta-analyses**
eTable 7. **Duval and Tweedie's trim-and-fill method to adjust for publication bias**
eTable 8. **Sex differences in the prevalence NPS for meta-analyses that excluded outliers**
eTable 9. **Sex differences in the severity of NPS for meta-analyses that excluded outliers**
eFigure 1. **Forest plots for meta-analyses prevalence specific NPS**
eFigure 2. **Forest plots for meta-analyses severity specific NPS**
eFigure 3. **Funnel plots for meta-analyses prevalence specific NPS**
eFigure 4. **Funnel plots for meta-analyses severity specific NPS**
eReferences

eTable 1 Search strategy literature search.

Embase.com – inception until February 12, 2021<br>('behavior'/de OR 'neuropsychiatry'/de OR 'neuropsychiatric inventory'/de OR 'apathy'/de OR 'agitation'/de OR 'restlessness'/de OR 'sleep'/exp OR 'aggression'/de OR 'anxiety'/de OR 'depression'/de OR 'delusion'/de OR 'hallucination'/de OR 'eating'/de OR 'euphoria'/de OR 'irritability'/de OR (((neuropsychiatr*) NEAR/3 (symptom* OR inventor*)) OR NPS OR apath* OR agitat* OR sleep* OR aggress* OR anxiet* OR anxious* OR depress* OR delus* OR hallucinat* OR eat* OR euphor* OR disinhibit* OR irritabilit* OR (irritable NEAR/3 mood*) OR ((aberrant*) NEAR/3 (motor*) NEAR/3 (behavior* OR behaviour*)) OR ((mild*) NEAR/3 (behav*) NEAR/3 (impairment*)) OR MBI OR BPSD):ab,ti,kw) AND ('Alzheimer disease'/de OR (Alzheimer* OR Alzeimer*):ab,ti,kw) AND ('gender and sex'/exp OR 'sex ratio'/de OR ((gender* OR sex*) NEAR/3 (differ* OR specific* OR factor* OR characterist* OR ratio*)):ab,ti,kw) NOT ((animal/exp OR animal*:de OR nonhuman/de) NOT ('human'/exp)) NOT [conference abstract]/lim
Medline ALL/PubMed – inception until February 12, 2021<br>(Behavior/ OR Neuropsychiatry/ OR Apathy/ OR Psychomotor Agitation/ OR Sleep/ OR Aggression/ OR Anxiety/ OR Depression/ OR Delusions/ OR Hallucinations/ OR Eating/ OR Euphoria/ OR Irritable Mood/ OR (((neuropsychiatr*) ADJ3 (symptom* OR inventor*)) OR NPS OR apath* OR agitat* OR sleep* OR aggress* OR anxiet* OR anxious* OR depress* OR delus* OR hallucinat* OR eat* OR euphor* OR disinhibit* OR irritabilit* OR ((aberrant*) ADJ3 (motor*) ADJ3 (behavior* OR behaviour*)) OR ((mild*) ADJ3 (behav*) ADJ3 (impairment*)) OR MBI OR BPSD).ab,ti,kf.) AND (Alzheimer Disease/ OR (Alzheimer* OR Alzeimer*).ab,ti,kf.) AND (Sex Characteristics/ OR Sex/ OR Sex Factors/ OR exp Sex Distribution/ OR Sex Differentiation/ OR Gender Identity/ OR ((gender* OR sex*) ADJ3 (differ* OR specific* OR factor* OR characterist*)).ab,ti,kf.) NOT (exp animals/ NOT humans/) NOT (letter* OR news OR comment* OR editorial* OR congres* OR abstract* OR book* OR chapter* OR dissertation abstract*).pt.
Web of Science – inception until February 12, 2021<br>TS=(((((neuropsychiatr*) NEAR/2 (symptom* OR inventor*)) OR NPS OR apath* OR agitat* OR sleep* OR aggress* OR anxiet* OR anxious* OR depress* OR delus* OR hallucinat* OR eat* OR euphor* OR disinhibit* OR irritabilit* OR (irritable NEAR/2 mood*) OR ((aberrant*) NEAR/2 (motor*) NEAR/2 (behavior* OR behaviour*)) OR ((mild*) NEAR/2 (behav*) NEAR/2 (impairment*)) OR MBI OR BPSD)) AND ((Alzheimer* OR Alzeimer*)) AND (((gender* OR sex*) NEAR/2 (differ* OR specific* OR factor* OR characterist* OR ratio*))) NOT ((animal* OR rat OR rats OR mouse OR mice OR murine OR dog OR dogs OR canine OR cat OR cats OR feline OR rabbit OR cow OR cows OR bovine OR rodent* OR sheep OR ovine OR pig OR swine OR porcine OR veterinar* OR chick* OR zebrafish* OR baboon* OR nonhuman* OR primate* OR cattle* OR goose OR geese OR duck OR macaque* OR avian* OR bird* OR fish*) NOT (human* OR patient* OR women OR woman OR men OR man))) AND DT=(Article)
Cochrane Central Register of Controlled Trials – inception until February 12, 2021<br>((((neuropsychiatr*) NEAR/3 (symptom* OR inventor*)) OR NPS OR apath* OR agitat* OR sleep* OR aggress* OR anxiet* OR anxious* OR depress* OR delus* OR hallucinat* OR eat* OR euphor* OR disinhibit* OR irritabilit* OR (irritable NEAR/3 mood*) OR ((aberrant*) NEAR/3 (motor*) NEAR/3 (behavior* OR behaviour*)) OR ((mild*) NEAR/3 (behav*) NEAR/3 (impairment*)) OR MBI OR BPSD):ab,ti,kw) AND ((Alzheimer* OR Alzeimer*):ab,ti,kw) AND (((gender* OR sex*) NEAR/3 (differ* OR specific* OR factor* OR characterist* OR ratio*)):ab,ti,kw
PsycINFO – inception until February 12, 2021<br>(Behavior/ OR Neuropsychiatry/ OR Apathy/ OR Agitation/ OR Sleep/ OR Aggressiveness/ OR Anxiety/ OR "Depression (Emotion) "/ OR Delusions/ OR Hallucinations/ OR Eating Behavior/ OR Euphoria/ OR Irritability/ OR (((neuropsychiatr*) ADJ3 (symptom* OR inventor*)) OR NPS OR apath* OR agitat* OR sleep* OR aggress* OR anxiet* OR anxious* OR depress* OR delus* OR hallucinat* OR eat* OR euphor* OR disinhibit* OR irritabilit* OR ((aberrant*) ADJ3 (motor*) ADJ3 (behavior* OR behaviour*)) OR ((mild*) ADJ3 (behav*) ADJ3 (impairment*)) OR MBI OR BPSD).ab,ti.) AND ("Alzheimer's Disease"/ OR (Alzheimer* OR Alzeimer*).ab,ti.) AND (Human Sex Differences/ OR Sex/ OR Gender Identity/ OR ((gender* OR sex*) ADJ3 (differ* OR specific* OR factor* OR characterist*)).ab,ti.) NOT ((animal.po. OR exp animals/) NOT human.po.) NOT (letter* OR news OR comment* OR editorial* OR congres* OR abstract* OR book* OR chapter* OR dissertation abstract*).pt.
Google Scholar – top 200 relevant references<br>“gender|sex difference|differences|factor|ratio” “neuropsychiatric symptoms”|apathy|agitation|sleep|aggression|anxiety|depression|delusion|hallucination|eating|euphoria|disinhibition|irritability Alzheimer

eTable 2. Study quality assessment according to Quality Assessment Tool for Observational Cohort and Cross-Sectional Studies

Author, year	1	2	3	4	5	6	7	8	9	10	11	12	13	14	Overall quality
Alberici et al., 2008[1]	Yes	No	NR	NR	No	NA	NA	NA	No	NA	Yes	NA	NA	NA	Poor
Álvarez et al., 2014[2]	Yes	No	NR	Yes	No	NA	NA	NA	No	NA	Yes	NA	NA	NA	Fair
Benoit et al., 2008[3]	Yes	No	NR	Yes	No	NA	NA	NA	No	NA	Yes	NA	NA	NA	Fair
Bliwise et al., 2011[4]	Yes	Yes	Yes	Yes	No	NA	NA	NA	No	NA	Yes	NA	NA	NA	Fair
Burns et al., 1990a[5]	No	Yes	Yes	No	No	NA	NA	NA	No	NA	Yes	NA	NA	NA	Poor
Burns et al., 1990b[6]	No	Yes	NR	No	No	NA	NA	NA	No	NA	Yes	NA	NA	NA	Poor
Chang et al., 2020[7]	Yes	No	NR	No	Yes	NA	NA	NA	No	NA	Yes	NA	NA	NA	Fair
Charernboon et al., 2014[8]	Yes	Yes	NR	No	No	NA	NA	NA	No	NA	Yes	NA	NA	NA	Fair
Chiu et al., 2012[9]	Yes	Yes	NR	Yes	No	NA	NA	NA	No	NA	Yes	NA	NA	NA	Fair
Colombo et al., 2018[10]	Yes	No	Yes	Yes	No	NA	NA	NA	No	NA	Yes	NA	NA	NA	Fair
Connors et al., 2018[11]	Yes	Yes	Yes	Yes	No	NA	NA	NA	No	NA	Yes	NA	NA	NA	Good
Del Prete et al., 2009[12]	Yes	Yes	NR	Yes	No	NA	NA	NA	No	NA	Yes	NA	NA	NA	Fair
Delano-Wood et al., 2008[13]	Yes	No	NR	Yes	No	NA	NA	NA	No	NA	Yes	NA	NA	NA	Fair
Derouesné et al., 1996[14]	Yes	Yes	NR	Yes	No	NA	NA	NA	No	NA	Yes	NA	NA	NA	Fair
Eustace et al., 2001[15]	Yes	No	NR	Yes	No	NA	NA	NA	No	NA	Yes	NA	NA	NA	Fair
Finger et al., 2017[16]	Yes	Yes	NR	Yes	No	NA	NA	NA	No	NA	Yes	NA	NA	NA	Fair
Gomez-Gallego et al., 2020[17]	Yes	No	NR	Yes	No	NA	NA	NA	No	NA	Yes	NA	NA	NA	Fair
Gormley et al., 1998a[18]	Yes	Yes	NR	Yes	No	NA	NA	NA	No	NA	Yes	NA	NA	NA	Fair
Gormley et al., 1998b[19]	Yes	Yes	NR	Yes	No	NA	NA	NA	No	NA	Yes	NA	NA	NA	Fair
Hall et al., 2014[20]	Yes	No	NR	Yes	No	NA	NA	NA	No	NA	Yes	NA	NA	NA	Fair
Ikezaki et al., 2020[21]	Yes	Yes	NR	Yes	No	NA	NA	NA	No	NA	Yes	NA	NA	NA	Fair
Hsieh et al., 2019[22]	Yes	Yes	NR	No	No	NA	NA	NA	No	NA	Yes	NA	NA	NA	Fair
Kabeshita et al., 2016[23]	Yes	Yes	Yes	Yes	Yes	NA	NA	NA	No	NA	Yes	NA	NA	NA	Good
Kaiser et al., 2014[24]	Yes	No	NR	Yes	No	NA	NA	NA	No	NA	Yes	NA	NA	NA	Fair
Karttunen et al. 2011[25]	Yes	Yes	NR	Yes	No	NA	NA	NA	No	NA	Yes	NA	NA	NA	Fair
Kaufer et al., 1998[26]	Yes	No	NR	Yes	No	NA	NA	NA	No	NA	Yes	NA	NA	NA	Fair
Kwak et al., 2013[27]	Yes	Yes	Yes	Yes	No	NA	NA	NA	No	NA	Yes	NA	NA	NA	Good
Landes et al., 2005[28]	Yes	No	NR	Yes	No	NA	NA	NA	No	NA	Yes	NA	NA	NA	Fair
Lee et al., 2017[29]	Yes	Yes	NR	Yes	No	NA	NA	NA	No	NA	Yes	NA	NA	NA	Fair
Leroi et al., 2003[30]	Yes	Yes	NR	Yes	No	NA	NA	NA	No	NA	Yes	NA	NA	NA	Fair
Levy et al., 1996[31]	Yes	No	NR	Yes	No	NA	NA	NA	No	NA	Yes	NA	NA	NA	Fair
Linszen et al., 2018[32]	Yes	Yes	Yes	Yes	No	NA	NA	NA	No	NA	Yes	NA	NA	NA	Good

Author, year	1	2	3	4	5	6	7	8	9	10	11	12	13	14	Overall quality
Liu et al., 1999[33]	Yes	Yes	Yes	No	No	NA	NA	NA	No	NA	Yes	NA	NA	NA	Fair
Matsuoka et al., 2018[34]	Yes	Yes	NR	Yes	No	NA	NA	NA	No	NA	Yes	NA	NA	NA	Fair
Migliorelli et al., 1995[35]	Yes	Yes	Yes	Yes	No	NA	NA	NA	No	NA	Yes	NA	NA	NA	Good
Moran M. et al., 2005[36]	Yes	No	NR	Yes	No	NA	NA	NA	No	NA	Yes	NA	NA	NA	Fair
Moran E.K. et al., 2008[37]	Yes	No	NR	Yes	No	NA	NA	NA	No	NA	Yes	NA	NA	NA	Fair
Müller-Thomsen et al., 2002[38]	Yes	No	NR	No	No	NA	NA	NA	No	NA	Yes	NA	NA	NA	Poor
Mulin et al., 2011[39]	Yes	No	Yes	Yes	No	NA	NA	NA	No	NA	Yes	NA	NA	NA	Fair
de Oliveira et al., 2017[40]	Yes	Yes	NR	Yes	No	NA	NA	NA	No	NA	Yes	NA	NA	NA	Fair
Ott et al., 1996[41]	Yes	No	NR	Yes	No	NA	NA	NA	No	NA	Yes	NA	NA	NA	Fair
Porta-Etessam et al., 2011[42]	Yes	No	Yes	Yes	Yes	NA	NA	NA	No	NA	Yes	NA	NA	NA	Good
Pusswald et al., 2015[43]	Yes	Yes	Yes	Yes	No	NA	NA	NA	No	NA	Yes	NA	NA	NA	Good
Rozzini et al., 2007[44]	Yes	Yes	Yes	No	No	NA	NA	NA	No	NA	Yes	NA	NA	NA	Fair
Shi et al., 2020[45]	Yes	Yes	NR	Yes	No	NA	NA	NA	No	NA	Yes	NA	NA	NA	Fair
Senanarong et al., 2004[46]	Yes	No	NR	Yes	Yes	NA	NA	NA	No	NA	Yes	NA	NA	NA	Fair
Sinforiani et al., 2010[47]	Yes	Yes	NR	Yes	No	NA	NA	NA	No	NA	Yes	NA	NA	NA	Fair
Slifer et al., 2009[48]	Yes	No	NR	No	No	NA	NA	NA	No	NA	Yes	NA	NA	NA	Poor
Spalletta et al., 2010[49]	Yes	Yes	NR	Yes	No	NA	NA	NA	No	NA	Yes	NA	NA	NA	Fair
Steffens et al., 2005[50]	Yes	Yes	NR	Yes	No	NA	NA	NA	No	NA	Yes	NA	NA	NA	Fair
Suma et al., 2018[51]	Yes	Yes	Yes	Yes	No	NA	NA	NA	No	NA	Yes	NA	NA	NA	Good
Toyota et al., 2007[52]	Yes	Yes	Yes	Yes	No	NA	NA	NA	No	NA	Yes	NA	NA	NA	Good
Treiber et al., 2008[53]	Yes	Yes	NR	Yes	No	NA	NA	NA	No	NA	Yes	NA	NA	NA	Fair
Troisi et al., 1996[54]	Yes	No	NR	No	No	NA	NA	NA	No	NA	Yes	NA	NA	NA	Poor
Tunnard et al., 2010[55]	Yes	No	NR	Yes	No	NA	NA	NA	No	NA	Yes	NA	NA	NA	Fair
Usman et al., 2010[56]	Yes	Yes	Yes	No	No	NA	NA	NA	No	NA	Yes	NA	NA	NA	Fair
Vilalta-Franch et al., 2013[57]	Yes	Yes	NR	Yes	No	NA	NA	NA	No	NA	Yes	NA	NA	NA	Fair
Xing et al., 2015[58]	Yes	No	NR	No	No	NA	NA	NA	No	NA	Yes	NA	NA	NA	Poor
Yang et al., 2017[59]	Yes	Yes	NR	Yes	No	NA	NA	NA	No	NA	Yes	NA	NA	NA	Fair
Yang et al., 2020[60]	Yes	Yes	NR	Yes	No	NA	NA	NA	No	NA	Yes	NA	NA	NA	Fair
Zeitzer et al., 2014[61]	Yes	No	NR	Yes	No	NA	NA	NA	No	NA	Yes	NA	NA	NA	Fair
Zhao et al., 2012[62]	No	No	NR	Yes	No	NA	NA	NA	No	NA	Yes	NA	NA	NA	Poor

Abbreviations: NR, not reported; NA, not applicable.

eTable 3. Characteristics of included studies

Study	Country	Sample	N total	N (%) females	N (%) males	Age M (SD)	Education M (SD)	MMSE M (SD)	Disease duration M (SD)	% *APOE-ε4* carriers	NPS	Outcome measure
Alberici et al., 2008[1]	Italy	*NR*	25	16 (64.0 %)	9 (36.0 %)	79.2 (7.2)	*NR*	16.0 (7.8)	*NR*	*NR*	Total score	NPI
Álvarez et al., 2014[2]	Spain	Memory clinic	250	194 (77.6 %)	56 (22.4 %)	75.0 (7.4)	*NR*	17.5 (4.1)	*NR*	47.2%	Apathy	NPI-item >0
Benoit et al., 2008[3]	France	Memory clinic	684	487 (71.0%)	197 (28.7%)	77.9 (6.8)	*NR*	20.0 (4.23)	1.1 (1.1)	*NR*	Apathy, depression	NPI-items >0
Bliwise et al., 2011[4]	USA	Memory clinic	4192	2,387 (56.9%)	1,805 (43.1%)	76.2 (9.4)	>College 57.5%	21.6 (15.0)	NR	*NR*	Night-time behaviors	NPI-Q-item >0
Burns et al., 1990a[5]	UK	Memory clinic, NH	174	139 (79.9%)	35 (20.1%)	80.5 (6.6)	*NR*	*NR*	*NR*	*NR*	Depression	GMSS >0
Burns et al., 1990b[6]	UK	Memory clinic, NH	178	141 (79.2%)	37 (20.8%)	80.5 (6.6)	*NR*	*NR*	*NR*	*NR*	Aggression	SGRC >0
Chang et al., 2020[7]	Taiwan	Memory clinic	463	259 (55,9%)	204 (44,1%)	70.4 (7.7)	7.1 (4.9)	18.5 (7.3)	*NR*	30.2%	Total score, 12 NPI-items	NPI severity x frequency scores
Charernboon et al., 2014[8]	Thailand	Memory clinic	62	39 (62.9%)	23 (37.1%)	76.0 (6.7)	*NR*	18.3 (5.9)	*NR*	*NR*	No. NPS	NPI
Chiu et al., 2012[9]	Taiwan	Memory clinic	302	203 (67.2%)	99 (32.8%)	76.8 (8.8)	5.0 (4.6)	15.3 (8.3)	2.7 (2.5)	*NR*	Depression	NIMH-dAD diagnosis
Colombo et al., 2018[10]	Italy	Memory clinic	221 mild	108 (48.9%)	113 (51.1%)	76.4 (7.0)[a]	≥High School 41.8%	Range 18–26	*NR*	*NR*	12 NPI-items	NPI-items >0
Colombo et al., 2018[10]	Italy	Memory clinic	254 mod.	166 (65.4%)	88 (34.7%)	76.4 (7.0)[a]	≥ High School 41.8%	Range 10–17	*NR*	*NR*	12 NPI-items	NPI-items >0

Study	Country	Sample	N total	N (%) females	N (%) males	Age M (SD)	Education M (SD)	MMSE M (SD)	Disease duration M (SD)	% APOE-ε4 carriers	NPS	Outcome measure
Connors et al., 2018[11]	Australia	Memory clinic	445	223 (50.1%)	222 (49.9%)	78.7 (7.3)	>Secondary 33.7%	21.1 (5.3)	1.6 (1.9)	*NR*	Hallucinations, delusions	NPI-items >0
Del Prete et al., 2009[12]	Italy	Memory clinic	53	36 (67.9%)	17 (32.1%)	74.0 (7.1)	6.2 (4.0)	19.3 (4.5)	2.0 (1.4)	47.2%	Depression, Anxiety	NPI-items >0
Delano-Wood et al., 2008[13]	USA	Memory clinic	323	160 (49.5%)	163 (50.5%)	72.6 (9.2)	13.0 (4.0)	20.4 (5.5)	3.8 (2.4)	67.5%	Depression	DSM-III-R DD diagnosis
Derouesné et al., 1996[14]	France	Memory clinic	135	74 (54.8%)	61 (45.2%)	69.3 (8.9)	*NR*	17.1 (6.5)	4.3 (2.3)	*NR*	Total score	PBQ
Eustace et al., 2001[15]	Ireland	Memory clinic	150	103 (68.7%)	47 (31.3%)	76.5 (8.3)	*NR*	19.3 (4.7)	*NR*	*NR*	Aggression	BEHAVE-AD >0
Finger et al., 2017[16]	Canada	Memory clinic	758	315 (41.6%)	443 (58.4%)	75.3 (6.8)	15.6 (2.8)	26.4 (3.4)	*NR*	26.1%	Disinhibition	NPI-Q or NPI-item >0
Gomez-Gallego et al., 2020[17]	Spain	Primary healthcare, day centers	196	119 (60.7%)	77 (39.3%)	77.6 (8.0)	4.5 (2.8)	17.1 (5.0)	*NR*	*NR*	9 NPI-items	Severity x frequency scores
Gormley et al., 1998a[18]	UK	Day center, inpatients	70	45 (64.0%)	25 (36.0%)	78.3 (6.7)	*NR*	12.7 (7.9)	*NR*	*NR*	Aggression	RAGE item 21 >0
Gormley et al., 1998b[19]	UK	Day center, inpatients	70	45 (64.0%)	25 (36,0%)	78.3 (6.7)	*NR*	12.7 (7.9)	*NR*	*NR*	Psychotic symptoms	BEHAVE-AD >0
Hall et al., 2014[20]	USA	Memory clinic	220	144 (72.0%)	76 (28.0%)	77.7 (6.9)	14.1 (3.2)	19.1 (6.1)	*NR*	59.0%	No. NPS	NPI-Q
Hsieh et al., 2020[22]	Taiwan	Memory clinic, NH	460	280 (60.9 %)	180 (39.1 %)	77.2 (7.9)	<6y 55.0% >6y 45.0%	17.4 (6.4)	*NR*	22.0%	12 NPI-items	Severity x frequency scores
Ikezaki et al., 2020[21]	Japan	Memory clinic	230	159 (69.1%)	71 (30.9%)	77.3 (7.9)	11.3 (2.3)	23.3 (1.9)	2.3 (1.6)	*NR*	Total score, 10 NPI-items, depression	NPI everity x frequency scores, GDS-15

Study	Country	Sample	N total	N (%) females	N (%) males	Age M (SD)	Education M (SD)	MMSE M (SD)	Disease duration M (SD)	% APOE-ε4 carriers	NPS	Outcome measure
Kabeshita et al., 2016[23]	Japan	Memory clinic	684	478 (69.9%)	206 (30.1%)	76.3 (8.7)	11.0 (2.9)	18.8 (5.3)	*NR*	*NR*	Night-time behaviors	NPI-item >0
Kaiser et al., 2014[24]	USA	Memory clinic	22 LO	7 (31.8%)	15 (68.2%)	80.3 (5.9)	*NR*	*NR*	3.5 (2.2)	*NR*	Anxiety	NPI-item >0
Karttunen et al., 2011[25]	Finland	Memory clinic	240	123 (51.3%)	117 (48.7%)	75.1 (6.6)	7.5 (*NR*)	21.5 (3.4)	3.3 (2.1)	*NR*	Delusions, AMB, depression	NPI-items >0, BDI total score
Kaufer et al., 1998[26]	USA	Memory clinic	85	41 (48.2%)	44 (51.8%)	75.9 (6.9)	*NR*	16.6 (7.7)	*NR*	*NR*	Total score	NPI
Kwak et al., 2013[27]	Korea	Memory clinic	230	142 (61.7%)	88 (38.3%)	74.7 (7.8)	8.9 (5.8)	19.2 (5.8)	2.9 (2.2)	*NR*	Delusions	NPI-item >0
Landes et al., 2005[28]	USA	Memory clinic	131	70 (53.4%)	61 (46.6%)	75.1 (8.1)	13.0 (3.3)	18.5 (6.9)	*NR*	*NR*	Apathy, depression	DAIR, BRDS total scores
Lee et al., 2017[29]	Korea	Memory clinic	66 mild	39 (57.4%)	27 (39.7%)	77.0 (7.0)	9.6 (4.6)	22.4 (2.5)	*NR*	*NR*	12 NPI-items	Severity x frequency scores
Lee et al., 2017[29]	Korea	Memory clinic	59 mod.	39 (66.1%)	20 (33.9%)	77.3 (7.5)	6.4 (5.1)	14.7 (2.4)	*NR*	*NR*	12 NPI-items	Severity x frequency scores
Leroi et al., 2003[30]	USA	Community based	201	138 (53.1%)	63 (46.9%)	84.6 (6.7)	12.4 (3.0)	15.9 (7.7)	4.7 (3.3)	33.0%	Psychosis	NPI delusions or hallucinations >0
Levy et al., 1996[31]	USA	Placebo arm of RCT	181	100 (55.0%)	81 (45.0%)	74.0% <76.0	≥College 50.0%	72.0% <23.0	*NR*	*NR*	Depression, agitation, psychosis	ADAS-Non-Cog >0
Linszen et al., 2018[32]	Netherlands	Memory clinic	1,227	629 (51.3%)	598 (48.7%)	66.6 (7.9)	NR	21.0 (17–24)[c]	NR	61.9%	Hallucinations	NPI-item >0
Liu et al., 1999[33]	Taiwan	Memory clinic	141	78 (55.3%)	63 (44.7%)	72.7 (8.7)	7.9 (5.8)	*NR*	*NR*	*NR*	Depression	DSM-III-R DD diagnosis

Study	Country	Sample	N total	N (%) females	N (%) males	Age M (SD)	Education M (SD)	MMSE M (SD)	Disease duration M (SD)	% APOE-ε4 carriers	NPS	Outcome measure
Matsuoka et al., 2018[34]	Japan	Memory clinic	63	44 (69.8%)	19 (30.2%)	79.0 (6.9)	*NR*	20.1 (4.2)	1.8 (1.4)	*NR*	Night-time behaviors	NPI-item >0
Migliorelli et al., 1995[35]	Argentina	Memory clinic	103	76 (73.8%)	27 (26.2%)	73.6 (7.5)	10.0 (4.9)	18.7 (6.5)	4.3 (2.5)	*NR*	Depression	DSM-III-R MD, dystemia diagnosis
Moran E.K. et al., 2008[37]	USA	Memory clinic	103	68 (66.0%)	35 (34.0%)	79.0 (6.0)	*NR*	16.5 (6.2)	*NR*	*NR*	Psychotic symptoms	BEHAVE-AD >0
Moran M. et al., 2005[36]	Ireland	Memory clinic	224	157 (70.1%)	67 (29.9%)	74.9 (7.7)	*NR*	18.3 (5.3)	*NR*	*NR*	Sleep disturbance	BEHAVE-AD >0
Mulin et al., 2011[39]	Multicenter (Europe, USA, Argentina)	Memory clinic	132	88 (66.7%)	44 (33.3%)	79.2 (6.5)	*NR*	18.3 (4.6)	*NR*	*NR*	Apathy	Apathy in AD diagnosis
Müller-Thomsen et al., 2002[38]	Germany	Memory clinic	137	93 (67.9%)	44 (32.1%)	71.3 (8.9)	9.3 (1.6)	18.1 (6.2)	3.2 (2.4)	60.5%	Depression	ICD-10 DD diagnosis
de Oliveira et al., 2017[40]	Brazil	Memory clinic	201	141 (70.1%)	60 (29.9%)	73.5 (6.3)	NR	NR	NR	53.7%	10 NPI-items	NPI-items >0, Severity x frequency scores
Ott et al., 1996[41]	USA	Memory clinic	125	75 (60.0%)	50 (40.0%)	74.7 (6.0)	12.1 (6.7)	18.3 (5.6)	2.9 (1.6)	*NR*	Total score, depression	DBDS, CSDD
Porta-Etessam et al., 2011[42]	Spain	Memory clinic, NH, day center	1,248	850 (68.1%)	398 (31.9%)	77.8 (6.7)	NR	15.7 (4.3)[d]	2.0 (1.3)	*NR*	Depression	CSDD >9
Pusswald et al. 2015[43]	Austria	Memory clinic	286	173 (60.5%)	113 (39.5%)	79.0 (73–84)[c]	8.5 (9–13)[c]	22.5 (19–25)[c]	2.0 (1–3)[c]	*NR*	Depression	GDS-15
Rozzini et al., 2007[44]	Italy	Memory clinic	135	99 (73.3%)	36 (26.7%)	75.5 (7.2)	5.9 (3.0)	20.9 (3.9)	2.2 (1.1)	*NR*	Depression	DSM-IV dAD diagnosis

Study	Country	Sample	N total	N (%) females	N (%) males	Age M (SD)	Education M (SD)	MMSE M (SD)	Disease duration M (SD)	% APOE-ε4 carriers	NPS	Outcome measure
Senanarong et al., 2004[46]	USA	Memory clinic	277	188 (68%)	89 (32%)	76.3 (8.0)	13.0 (3.6)	19.2 (6.5)	NR	*NR*	Agitation	NPI-items >0, Severity x frequency scores
Shi et al., 2020[45]	China	Memory clinic	158	86 (54.4%)	72 (45.6%)	73.1 (5.6)	*NR*	23.8 (1.8)	*NR*	*NR*	Any NPS	NPI-Q >0
Sinforiani et al., 2010[47]	Spain	Memory clinic	600	386 (64.3%)	214 (35.7%)	75.1 (6.5)	5.5 (4.3)	19.2 (3.5)	*NR*	*NR*	Total score	NPI
Slifer et al., 2009[48]	USA	Memory clinic	528	337 (63.8%)	191 (36.2%)	72.2 (6.2)	*NR*	*NR*	*NR*	48.7%	Depression	GDS >9
Spalletta et al., 2010[49]	Italy	Memory clinic	1,015	723 (71.2%)	292 (28.8%)	74.6 (0.2)	6.2 (0.1)	18.3 (0.2)	2.7 (0.1)	*NR*	12 NPI-items	Severity x frequency scores
Steffens et al., 2005[50]	USA	Community based	149	24 (16.1%)	125 (83.9%)	77.3 (5.0)[e]	*NR*	*NR*	*NR*	*NR*	12 NPI-items	NPI-items >0, Severity x frequency scores
Suma et al., 2018[51]	Japan	Memory clinic	853	578 (67.8%)	275 (32.2%)	79.0 (6.5)	10.3 (2.6)	18.8 (4.1)	*NR*	*NR*	Eating behaviors	CNAQ <29
Toyota et al., 2007[52]	Japan	Memory clinic	261 LO	181 (69.3%)	80 (30.7%)	78.5 (5.1)	9.5 (2.3)	19.0 (6.0)	3.2 (2.4)	*NR*	Delusions	NPI-item >0
Toyota et al., 2007[52]	Japan	Memory clinic	46 EO	22 (47.8%)	24 (52.2%)	58.8 (5.0)	11.8 (2.7)	17.4 (6.3)	3.5 (2.0)	*NR*	Delusions	NPI-item >0
Treiber et al., 2008[53]	USA	Community based	254	173 (68.1%)	81 (31.9%)	85.7 (6.6)	13.3 (2.9)	*NR*	1.9 (1.3)	*NR*	Disinhibition	NPI Severity x frequency scores
Troisi et al., 1996[54]	Italy	Memory clinic	42	25 (59.5%)	17 (40.5%)	76.2 (6.1)	6.6 (4.7)	14.2 (5.9)	*NR*	*NR*	Depression	HRSD-PSY subscore
Tunnard et al., 2011[55]	UK	Memory clinic	111	76 (68.5%)	35 (31.5%)	75.2 (6.2)	8.1 (4.0)	20.8 (4.8)	4.0 (2.9)	*NR*	Apathy	NPI-item >0

Study	Country	Sample	N total	N (%) females	N (%) males	Age M (SD)	Education M (SD)	MMSE M (SD)	Disease duration M (SD)	% APOE-ε4 carriers	NPS	Outcome measure
Usman et al., 2010[56]	Pakistan	Memory clinic	76	42 (55.3%)	34 (44.7%)	67.7 (6.7)	>12y 10.5%	68.4% <20.0	*NR*	*NR*	Depression	HRSD >7
Vilalta-Franch et al., 2013[57]	Spain	Memory clinic	491	348 (70.9%)	143 (29.1%)	75.2 (6.6)	5.9 (3.7)	*NR*	2.7 (2.3)	*NR*	Psychosis	PoAD diagnosis
Xing et al., 2015[58]	China	Memory clinic	158 mild	84 (53.2%)	74 (46.8%)	70.9 (9.6)	7.6 (5.2)	17.5 (5.2)	*NR*	34.8%	12 NPI-items	NPI-items >0
Xing et al., 2015[58]	China	Memory clinic	157 mod.	92 (58.6%)	65 (41.4%)	67.7 (10.4)	6.1 (4.7)	12.3 (5.5)	*NR*	39.4%	12 NPI-items	NPI-items >0
Yang et al., 2017[59]	China	Community based	104	75 (72.1%)	29 (27.9%)	71.8 (60–85)	≥Secondary 15.4%	21.3 (4.8)[f]	*NR*	*NR*	Depression	GDS-30 total score
Yang et al., 2020[60]	China	Memory clinic	158	84 (53.2%)	74 (46.8%)	74.1 (5.8)	*NR*	14.5 (7.6)	*NR*	*NR*	Depression	HRSD ≥17
Zeitzer et al., 2013[61]	France	Memory clinic	92	46 (50%)	46 (50%)	77.9 (6.6)	*NR*	21.0 (5.5)	*NR*	*NR*	Apathy	NPI-item >2
Zhao et al., 2012[62]	China	Memory clinic	83	40 (48.2%)	43 (51.8%)	74.0 (8.3)	8.7 (4.0)	18.0 (7.7)	3.7 (3.1)	*NR*	Apathy	NPI-item >0

Abbreviations: MMSE, Mini-mental state examination; NPS, neuropsychiatric symptoms; NR, not reported; USA, United States of America; UK, United Kingdom; EO, early-onset; LO, late-onset; AMB, aberrant motor behaviors; NPI, Neuropsychiatric Inventory; NPI-Q, Neuropsychiatric Inventory Questionnaire; GMSS, Geriatric Mental State Schedule; SGRC, Stockton Geriatric Rating Scale; ICD-9, International Classification of Diseases; DD, depressive disorder; NIMH-dAD, National Institute of Mental Health criteria for depression in Alzheimer's disease (Olin et al. [2002] Am J Geriatr Psychiatry; DSM, Diagnostic and Statistical Manual of Mental Disorders; PBQ, Psychobehavioral Questionnaire; BEHAVE-AD,Behavioural pathology in Alzheimer's disease; RAGE, Rating scale for aggressive behaviour in the elderly; BDI, Beck Depression Inventory; DAIR,Dementia Apathy Interview and Rating; BRSD, Behavior Rating Scale for Dementia; ADAS-Non-Cog, non-cognitive section of the Alzheimer's disease Assessment Scale; DBDS, Dementia Behavior Disturbance Scale; CSDD, Cornell Scale for Depression in Dementia; GDS, Geriatric Depression Scale; CNAQ, Council of Nutrition Appetite Questionnaire; HRSD-PSY,

Intrapsychic subscore of the Hamilton Rating Scale for Depression; PoAD, psychosis in Alzheimer's disease (Jeste & Finkel [2000] Am J Geriatr).

[a] M(SD) of total sample including mild and moderate AD

[b] Studies reported corrected effect sizes

[c] Median (interquartile range)

[d] Baseline MMSE scores of subsample of patients who had six months follow-up assessments available (N=852/N=1248).

[e] M (SD) age of total dementia population (N=211) of which 149 patients had AD

[f] M(SD) of the Montreal Cognitive Assessment (MoCA)

eTable 4. Meta-regression analyses on the prevalence of specific neuropsychiatric symptoms in Alzheimer's disease dementia

NPS	K	Clinically relevance (presence vs relevance)	Method of assessment (proxy vs self-report)	NPI vs non-NPI	Mean age	Mean education in years	Mean MMSE score	Mean disease duration in years	%*APOE*-E4 carriers
Aberrant motor behaviors	7	*NR*	*NR*	*NR*	*k*=7, *QM*=0.22, *p*=0.64, estimate=-0.03	*NA*	*NA*	*NR*	*NR*
Agitation	11	*NA*	*NR*	*k*=11, *QM*=0.98, *p*=0.33, NPI OR=0.67 [0.36–1.55], nonNPI OR=0.99 [0.63–1.55]	*k*=10, *QM*=3.02, *p*=0.08, estimate=-0.08	*NA*	*k*=7, *QM*=0.01, *p*=0.93, estimate=0.01	*NR*	*NR*
Anxiety	8	*NR*	*NR*	*NR*	*k*=8, *QM*=0.54, *p*=0.46, estimate=-0.09	*NA*	*NA*	*NR*	*NA*
Apathy	12	*NA*	*NR*	*NA*	*k*=12, *QM*=0.02, *p*=0.89, estimate=-0.01	*NA*	*k*=10, *QM*=0.53, *p*=0.47, estimate=-0.05	*NA*	*NA*
Delusions	12	*NR*	*NR*	*NR*	*k*=12, *QM*=0.00, *p*=0.99, estimate=0.00	*k*=7, *QM*=1.65, *p*=0.20, estimate=0.10	*k*=10, *QM*=0.01, *p*=0.92, estimate=0.00	*k*=6, *QM*=5.19, *p*=0.02, estimate=0.32	*NA*
Depressive symptoms	20	*k*=20, *QM*=0.09, *p*=0.76, presence OR=1.66 [1.21–2.28], clinical relevance OR=1.55 [1.13–2.11]	*k*=20, *QM*=0.05, *p*=0.83, Proxy OR=1.63 [1.25–2.12], Self-report OR=1.54 [1.00–2.37]	*k*=20, *QM*=0.09, *p*=0.77, NPI OR=1.67 [1.17–2.39], nonNPI OR=1.56 [1.17–2.08]	*k*=19, *QM*=0.91, *p*=0.34, estimate=-0.04	*k*=9, *QM*=0.00, *p*=0.95, estimate=0.01	*k*=13, *QM*=0.16, *p*=0.69, estimate=0.02	*k*=8, *QM*=0.30, *p*=0.59, estimate= 0.13	*k*=7, *QM*=0.04, *p*=0.84, estimate= 0.00
Disinhibition	8	*NR*	*NR*	*NR*	*k*=8, *QM*=8.20, *p*=0.004, estimate=-0.11	*NA*	*NA*	*NR*	*NA*

**NPS**	**K**	**Clinically relevance (presence vs relevance)**	**Method of assessment (proxy vs self-report)**	**NPI vs non-NPI**	**Mean age**	**Mean education in years**	**Mean MMSE score**	**Mean disease duration in years**	**%*APOE*-E4 carriers**
Euphoria	6	*NR*	*NR*	*NR*	*k*=6, *QM*=2.87, *p*=0.09, estimate=0.14	*NA*	*NA*	*NR*	*NR*
Halluci-nations	9	*NR*	*NR*	*NR*	*k*=9, *QM*=0.12, *p*=0.73, estimate=0.01	*NA*	*k*=6, *QM*=0.01, *p*=0.92, estimate=0.01	*NR*	*NA*
Irritability	6	*NR*	*NR*	*NR*	*k*=6, *QM*=1.09, *p*=0.30, estimate=-0.08	*NA*	*NA*	*NR*	*NR*
Sleep disturbances	8	*NR*	*NR*	*NR*	*NA*	*NA*	k=8, *QM*=0.11, *p*=0.74, estimate=-0.02	*NR*	*NR*

Abbreviations: NR, not reported; NA, not available due to the low number of studies.
Meta-regression analyses were not possible for eating behaviors, psychotic symptoms, and studies reporting on ≥1 NPS due to the low number of studies available.

eTable 5. Meta-regression analyses on severity of specific of specific neuropsychiatric symptoms in Alzheimer's disease dementia

NPS	k	Method of assessment (proxy vs self-report)	NPI vs non-NPI	Mean age of patients in years	Mean education in years	Mean MMSE score	Mean disease duration in years	%APOE E4 carriers
Aberrant motor behaviors	10	*NR*	*NA*	*k*=10, *QM*=0.00, *p*=0.96, estimate= 0.00	*k*=7, *QM*=0.31, *p*=0.58, estimate= -0.09	*k*=8, *QM*=0.13, *p*=0.72, estimate= 0.03	*NA*	*NA*
Agitation	12	*NR*	*NA*	*k*=12, *QM*=0.12, *p*=0.73, estimate= -0.02	*k*=8, *QM*=0.63, *p*=0.43, estimate= -0.09	*k*=10, *QM*=0.00, *p*=0.99, estimate= 0.00	*NA*	*NA*
Anxiety	11	*NR*	*NA*	*k*=11, *QM*=0.00, *p*=0.99, estimate= 0.00	*k*=7, *QM*=0.77, *p*=0.38, estimate= -0.48	*k*=9, *QM*=0.00, *p*=0.95, estimate= 0.02	*NA*	*NA*
Apathy	12	*NR*	*NA*	*k*=12, *QM*=0.04, *p*=0.83, estimate= 0.01	*k*=8, *QM*=0.40, *p*=0.52, estimate= 0.05	*k*=10, *QM*=0.01, *p*=0.91, estimate= -0.01	*NA*	*NA*
Delusions	11	*NR*	*NA*	*k*=11, *QM*=0.13, *p*=0.72, estimate= 0.02	*k*=7, *QM*=0.12, *p*=0.73, estimate= -0.06	*k*=9, *QM*=0.30, *p*=0.58, estimate= 0.04	*NA*	*NA*
Depressive symptoms	17	*k*=17, *QM*=0.39, *p*=0.54, proxy *g*=0.61 [-0.01–1.22], self-report *g*=0.29 [-0.48–1.07]	*k*=17, *QM*=0.39, *p*=0.54, NPI *g*=0.61 [-0.01–1.22], nonNPI *g*=0.29 [-0.48–1.07]	*k*=17, *QM*=0.03, *p*=0.86, estimate= -0.01	*k*=11, *QM*=0.93, *p*=0.33, estimate= -0.14	*k*=15, *QM*=0.05, *p*=0.82, estimate= -0.02	*NA*	*NA*
Disinhibition	11	*NR*	*NA*	*k*=11, *QM*=0.42, *p*=0.52, estimate= -0.03	*k*=7, *QM*=1.89, *p*=0.17, estimate= 0.14	*k*=9, *QM*=0.21, *p*=0.65, estimate= 0.02	*NA*	*NA*
Eating behavior	6	*NA*	*NA*	*k*=6, *QM*=0.99, *p*=0.32, estimate= 0.01	*NA*	*k*=6, *QM*=2.81, *p*=0.09, estimate= 0.04	*NA*	*NA*
Euphoria	11	*NR*	*NA*	*k*=11, *QM*=0.06, *p*=0.81, estimate= 0.01	*k*=7, *QM*=0.42, *p*=0.52, estimate= -0.06	*k*=9, *QM*=0.12, *p*=0.73, estimate= 0.02	*NA*	*NA*
Halluci-nations	11	*NR*	*NA*	*k*=11, *QM*=0.05, *p*=0.83, estimate= -0.01	*k*=7, *QM*=1.93, *p*=0.16, estimate= -0.22	*k*=9, *QM*=0.11, *p*=0.74, estimate= -0.03	*NA*	*NA*

**NPS**	**k**		**Assessment (NPI vs non-NPI)**	**Mean age of patients in years**	**Mean education in years**	**Mean MMSE score**	**Mean disease duration in years**	**%APOE E4 carriers**
Irritability	11	*NR*	*NA*	*k*=11, *QM*=0.00, *p*=0.97, estimate= 0.00	*k*=7, *QM*=0.01, *p*=0.93, estimate= 0.00	*k*=9, *QM*=0.16, *p*=0.68, estimate= -0.01	*NA*	*NA*
Sleep disturbances	6	*NR*	*NA*	*k*=6, *QM*=0.04, *p*=0.83, estimate= 0.00	*NA*	*k*=6, *QM*=4.40, *p*=0.04, estimate= 0.05	*NA*	*NA*
Total score NPS measure	14	*NR*	*NA*	*k*=14, *QM*=0.00, *p*=0.99, estimate= 0.00	*k*=7, *QM*=1.04, *p*=0.31, estimate= -0.17	*k*=12, *QM*=0.03, *p*=0.87, estimate= 0.02	*NA*	*NA*

Abbreviations: NR, not reported; NA, not available due to the low number of studies.

Meta-regression analyses were not possible for psychotic symptoms due to the low number of studies available.

eTable 6. Publication bias measures for all meta-analyses conducted

	k	Begg's rank test (τ)	P value	Egger's regression test (t)	P value
**NPS prevalence**					
Aberrant motor behaviors (excluding outlier)	6	0.29	0.78	-0.07	1.00
Agitation (excluding outlier)	10	-0.56	0.03	-2.82	0.02
Anxiety	8	-0.21	0.55	-0.63	0.55
Apathy	12	-0.36	0.12	-1.13	0.28
Any NPS (excluding outlier)	4	-0.33	0.75	-0.36	0.76
Delusions	12	0.33	0.15	1.74	0.11
Depressive symptoms	20	0.20	0.23	3.49	<0.01
Disinhibition	8	0.00	1.00	0.29	0.78
Eating behavior	5	0.20	0.82	-0.06	0.96
Euphoria	6	0.07	1.00	-0.14	0.90
Hallucinations	9	0.50	0.08	1.68	0.14
Irritability (excluding outlier)	5	-0.20	0.82	-1.44	0.24
Psychotic symptoms (excluding outlier)	4	-0.33	0.75	-0.32	0.78
Sleep disturbances	8	-0.14	0.72	-0.05	0.96
**NPS severity**					
Aberrant motor behaviors (excluding outlier)	9	-0.22	0.48	-0.72	0.50
Agitation (excluding outlier)	11	-0.16	0.54	-063	0.54
Anxiety (excluding outlier)	10	0.11	0.73	-0.03	0.98
Apathy (excluding outlier)	11	0.24	0.36	0.62	0.55
Delusions (excluding outlier)	10	-0.20	0.48	-0.77	0.46
Depressive symptoms (excluding outlier)	16	-0.03	0.89	0.18	0.86
Disinhibition (excluding outlier)	10	-0.29	0.29	-0.96	0.36
Eating behavior	6	-0.07	1.00	-0.07	0.94
Euphoria (excluding outlier)	10	-0.07	0.86	0.16	0.88
Hallucinations (excluding outlier)	10	-0.20	0.48	-0.29	0.78
Irritability	11	-0.27	0.28	-0.76	0.47
Total score NPS measure (excluding outlier)	13	-0.18	0.44	-1.27	0.23
Sleep disturbances	8	-0.14	0.72	-0.05	0.96

Abbreviations: k, number of studies; NPS, neuropsychiatric symptoms.

eTable 7. Duval and Tweedie's trim-and-fill method to adjust for potential publication bias

**Meta-analysis**	***k***	***OR* [95% CI][a]**	***z*-statistic**	***P***	***Q*-statistic**	***P Q*-statistic**	**I^2-statistic**
Prevalence agitation	10	1.00 [0.75, 1.35]	0.01	0.99	16.63	0.06	46.06
Trim-and-fill	13	1.20 [0.87, 1.66]	1.12	0.26	29.06	0.004	60.56
Prevalence depressive symptoms	20	1.60 [1.28, 1.98]	4.20	<0.001	51.99	<0.001	58.19
Trim-and-fill	21	1.55 [1.25, 1.94]	3.90	< 0.001	56.11	< 0.001	59.14

Abbreviations: k, number of studies; OR, odds ratio; NPS, neuropsychiatric symptoms.

[a] OR=1 no sex differences, OR>1 female sex associated with NPS; OR<1 male sex associated with NPS.

eTable 8. Sex differences in the prevalence of neuropsychiatric symptoms for meta-analyses that excluded outliers

NPS	*k*	*OR* [95% CI][a]	*z*-statistic	*P*	*Q*-statistic	*P Q*-statistic	I^2-statistic
Aberrant motor behaviors	6	1.47 [1.09, 1.98]	2.53	0.01	2.51	0.78	0.00
including outlier[25]	7	1.21 [0.79, 1.84]	0.87	0.39	14.16	0.03	57.41
Agitation	10	1.00 [0.75, 1.35]	0.01	0.99	16.63	0.06	46.06
including outlier[6]	11	0.87 [0.60, 1.25]	-0.77	0.44	28.57	0.002	66.49
Any NPS	4	1.35 [0.78–2.35]	1.07	0.28	4.01	0.25	32.74
including outlier[50]	5	1.06 [0.56, 2.01]	0.17	0.86	8.73	0.07	57.49
Irritability	5	1.14 [0.83, 1.56]	0.80	0.43	6.11	0.19	0.00
including outlier[40]	6	0.90 [0.55, 1.46]	-0.44	0.66	12.86	0.02	61.62
Psychotic symptoms	4	1.62 [1.12, 2.33]	2.56	<0.01	1.98	0.58	0.00
including outlier[19]	5	1.17 [0.60, 2.32]	0.46	0.64	12.74	0.01	73.02

Abbreviations: k, number of studies; OR, odds ratio; NPS, neuropsychiatric symptoms.

[a] OR=1 no sex differences, OR>1 female sex associated with NPS; OR<1 male sex associated with NPS.

eTable 9. Sex differences in the severity of neuropsychiatric symptoms for meta-analyses that excluded outliers

NPS	k	*Hedges' g* [95% CI][a]	*z*-statistic	*P*	*Q*-statistic	*P Q*-statistic	I^2-statistic
Aberrant motor behaviors	9	0.17 [0.08, 0.26]	3.56	<0.001	3.25	0.92	0.00
including outlier[49]	10	0.35 [-0.04, 0.74]	1.76	0.08	372.89	<0.001	95.21
Agitation	11	0.01 [-0.07, 0.10]	0.26	0.79	12.53	0.25	3.12
including outlier[49]	12	0.16 [-0.20, 0.52]	0.87	0.39	471.66	<0.001	95.45
Anxiety	10	0.11 [0.00, 0.22]	1.98	0.05	13.27	0.01	25.15
including outlier[49]	11	0.73 [-0.50, 1.97]	1.17	0.24	1544.28	<0.001	99.50
Apathy	11	-0.10 [-0.18, -0.01]	-2.25	0.02	5.00	0.89	0.00
including outlier[49]	12	-0.21 [-0.47, 0.05]	-1.61	0.11	244.64	<0.001	90.71
Depressive symptoms	16	0.24 [0.14, 0.34]	4.59	<0.001	30.15	0.02	44.29
including outlier[49]	17	0.49 [0.05, 0.96]	2.00	0.05	1140.34	<0.001	97.85
Delusions	10	0.19 [0.04, 0.34]	2.53	0.01	19.99	0.02	58.78
including outlier[49]	11	0.34 [-0.03, 0.72]	1.83	0.07	381.71	<0.001	95.17
Disinhibition	10	0.08 [-0.05, 0.21]	1.16	0.25	17.01	0.05	46.48
including outlier[49]	11	-0.06 [-0.30, 0.20]	-0.43	0.67	179.25	<0.001	89.63
Euphoria	10	0.00 [-0.10, 0.10]	-0.04	0.97	8.10	0.52	14.55
including outlier[49]	11	0.11 [-0.11, 0.33]	0.96	0.33	144.78	<0.001	86.49
Hallucinations	10	0.07 [-0.13, 0.26]	0.65	0.51	36.63	<0.001	77.20
including outlier[49]	11	0.25 [-0.15, 0.65]	1.20	0.23	452.69	<0.001	95.87
Total score NPS measure	13	0.04 [-0.04, 0.12]	1.03	0.31	7.54	0.82	0.00
including outlier[49]	14	0.29 [-0.24, 0.83]	1.07	0.29	1021.77	<0.001	97.89

Abbreviations: k, number of studies; OR, odds ratio; NPS, neuropsychiatric symptoms.

[a] Hedges' *g* >0 female sex associated with NPS; Hedges' *g* <0 male sex associated with NPS

eFigure 1. Forest plots meta-analyses prevalence specific NPS

A) Forest plot prevalence agitation

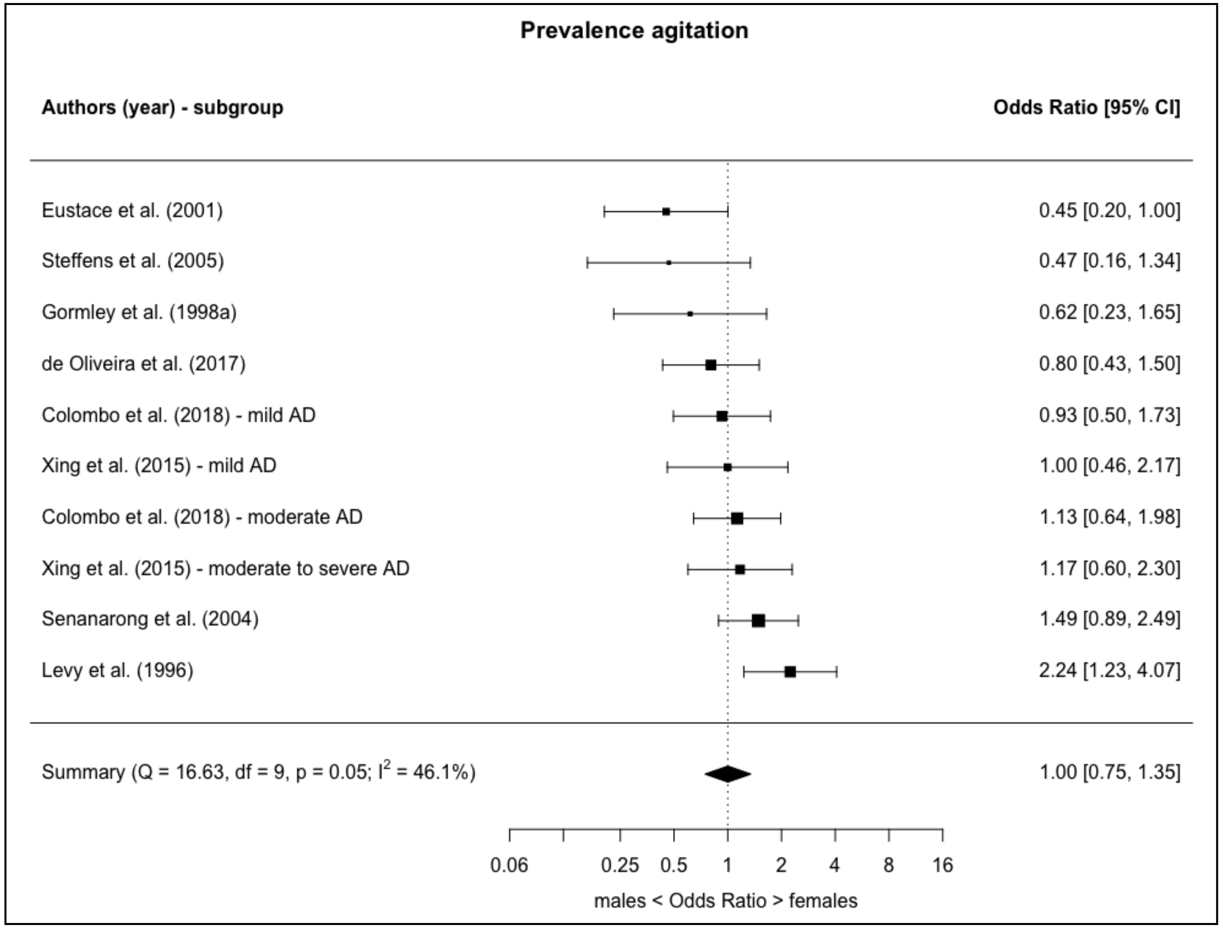

B) Forest plot prevalence anxiety

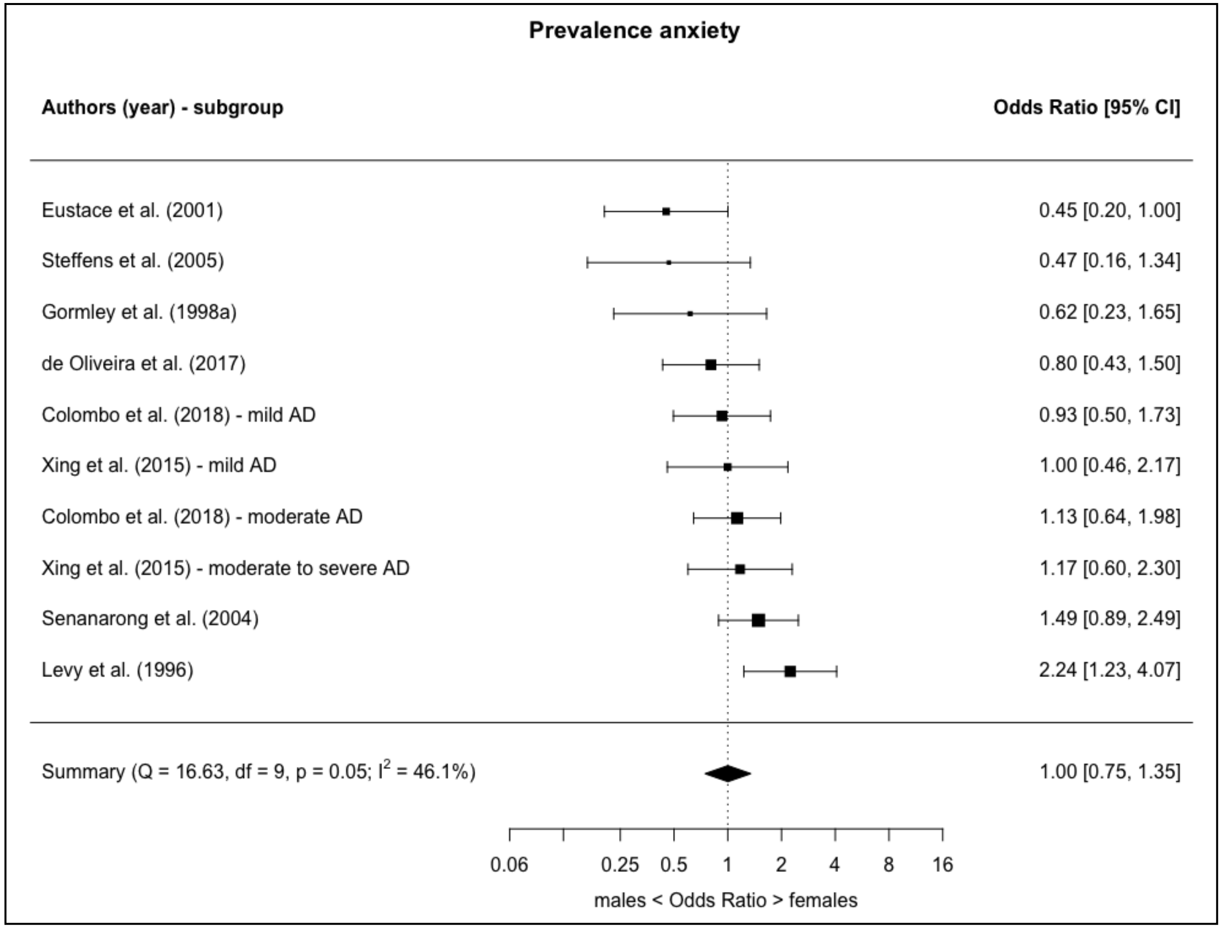

C) Forest plot prevalence apathy

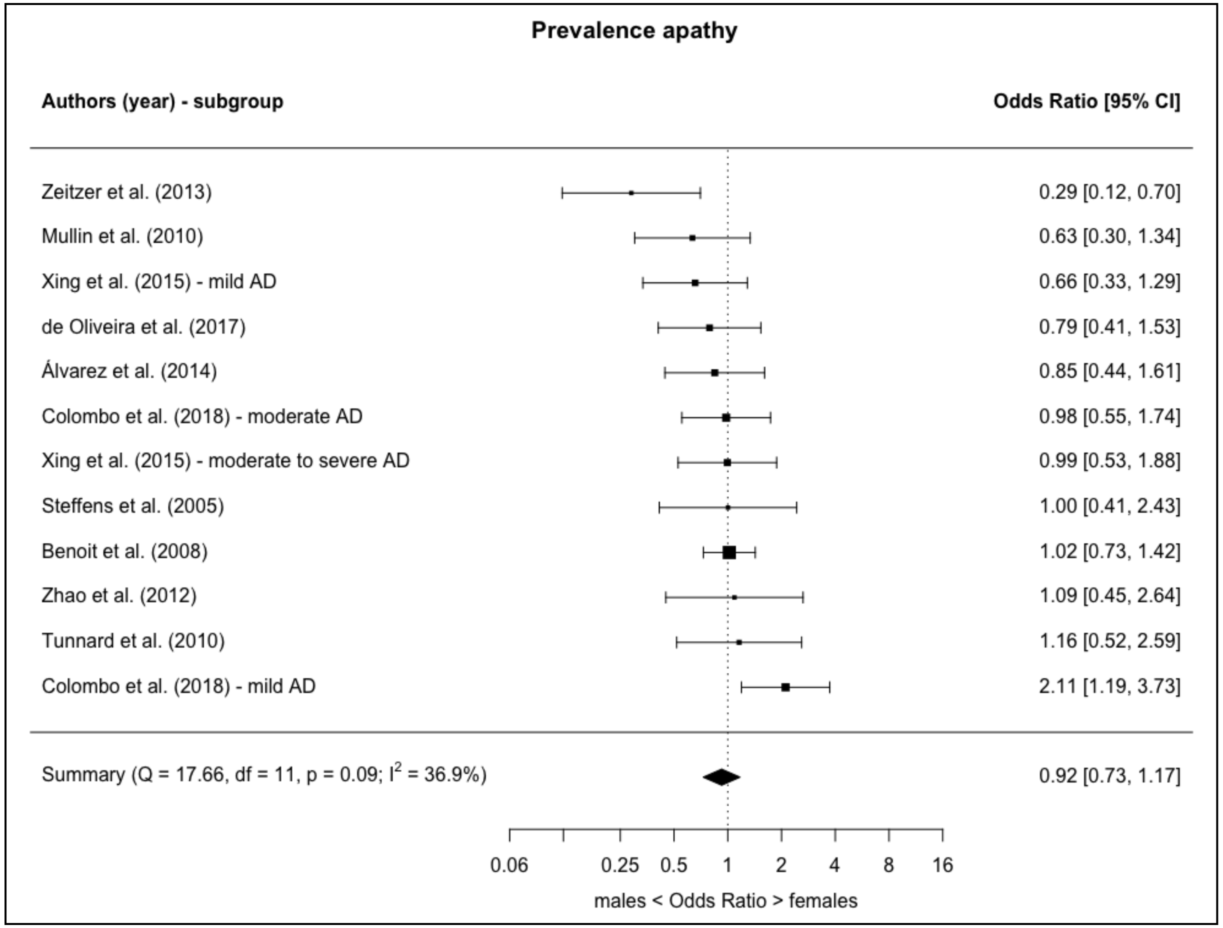

D) Forest plot prevalence disinhibition

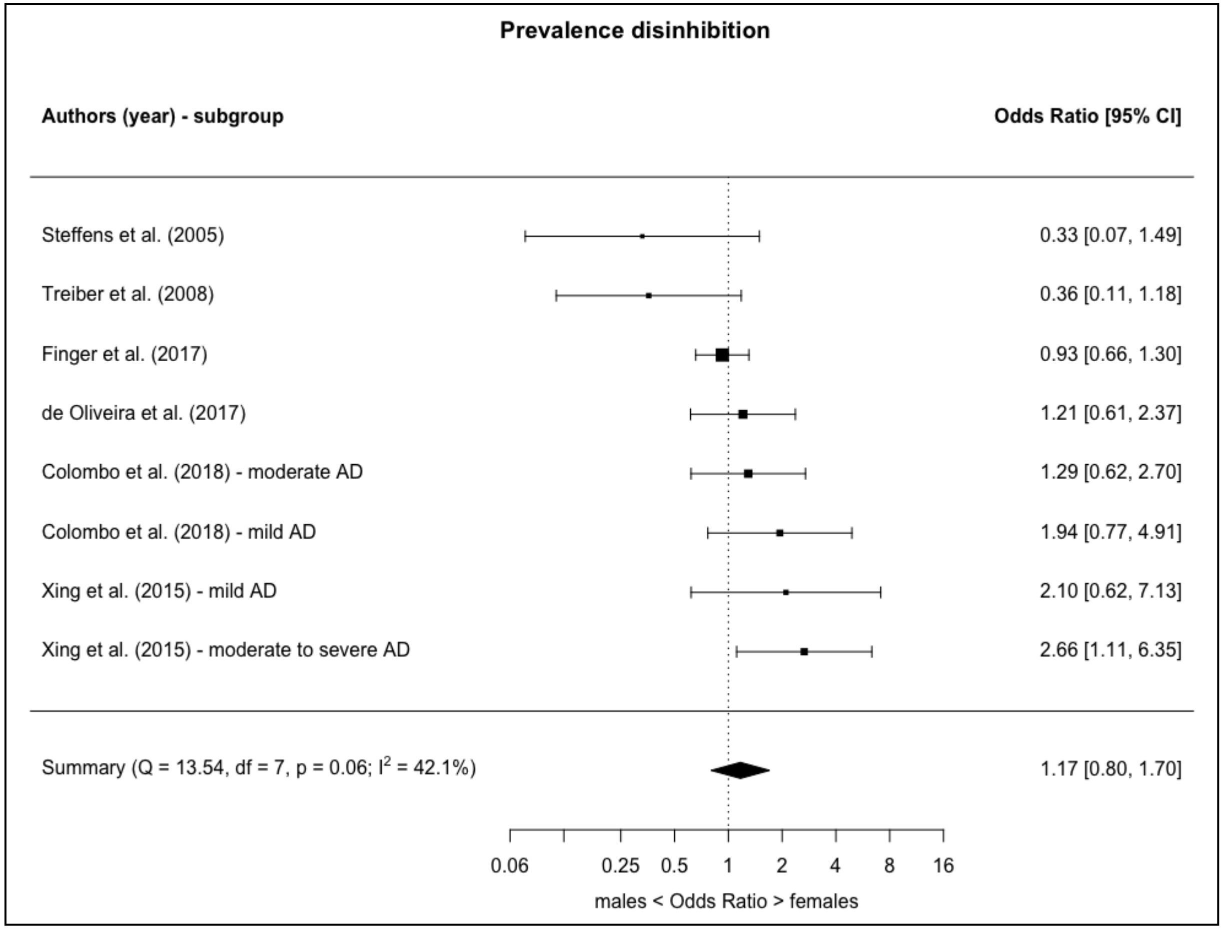

E) Forest plot prevalence eating behavior

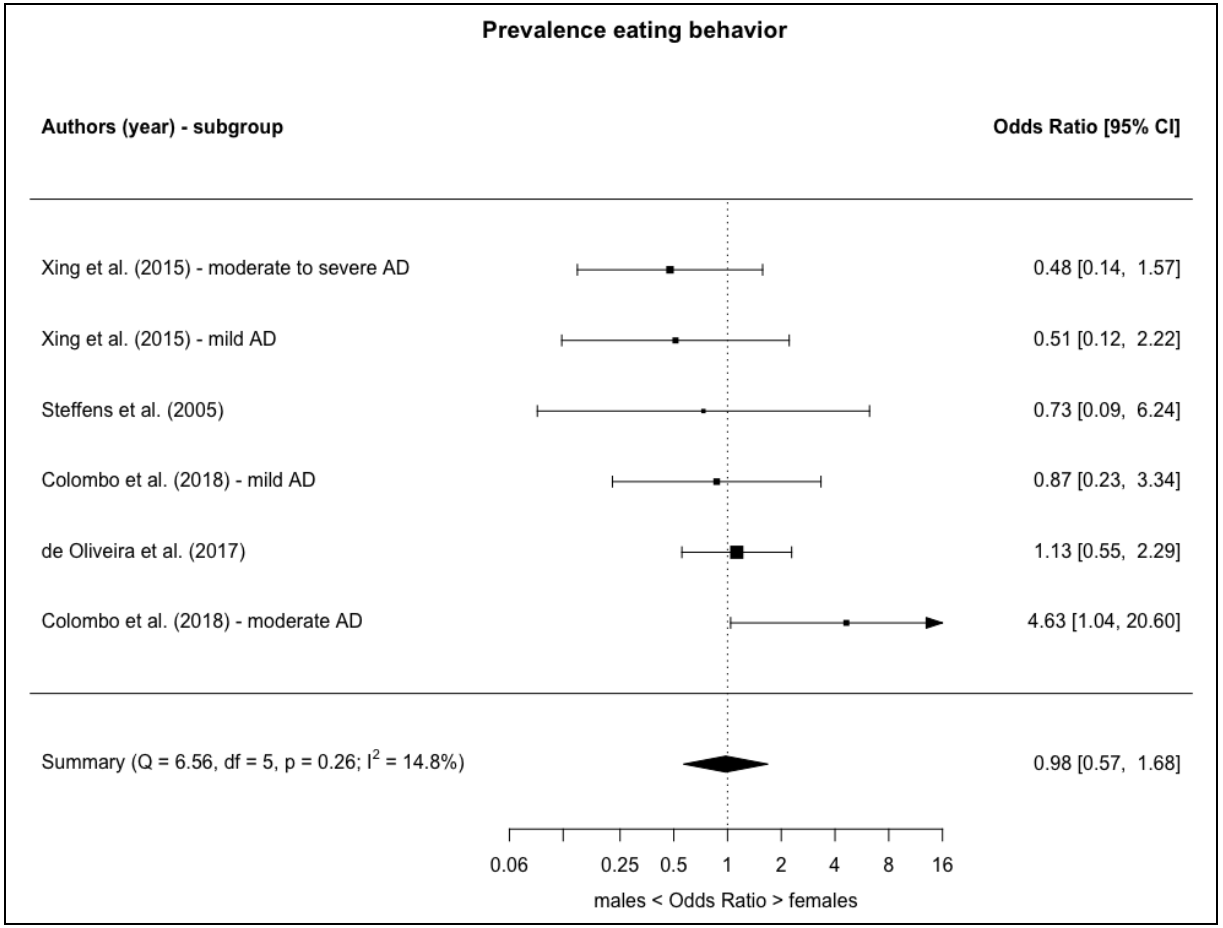

F) Forest plot prevalence euphoria

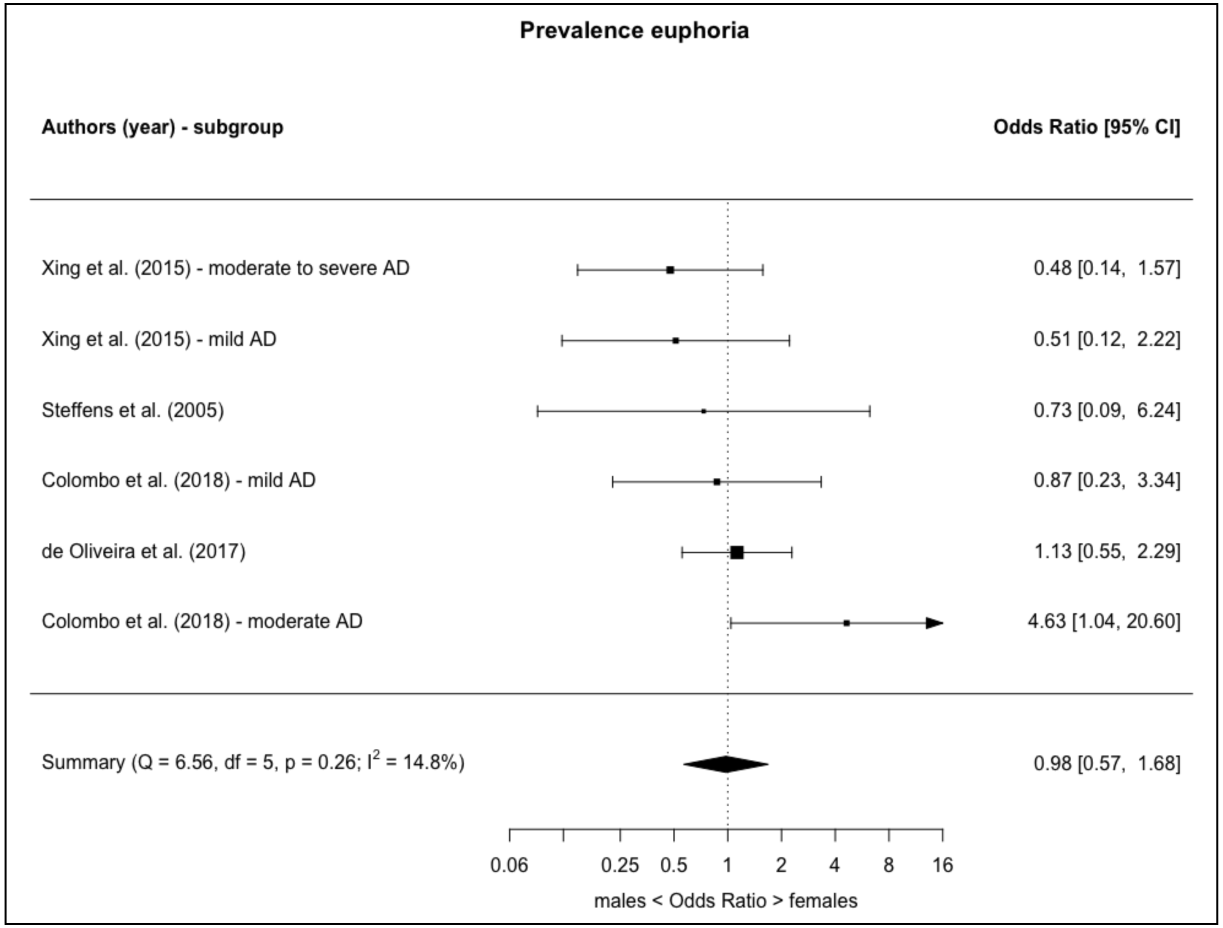

G) Forest plot prevalence hallucinations

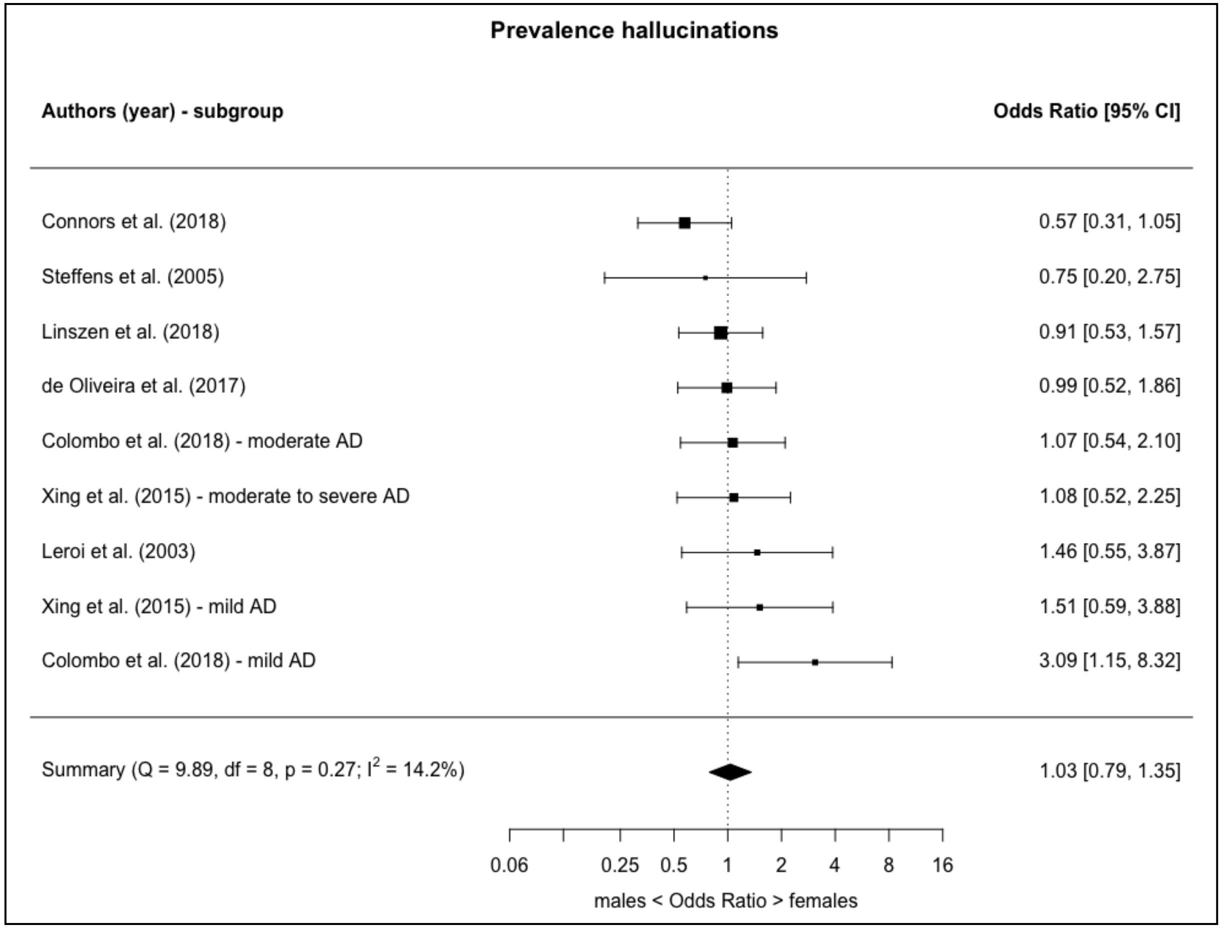

H) Forest plot prevalence irritability

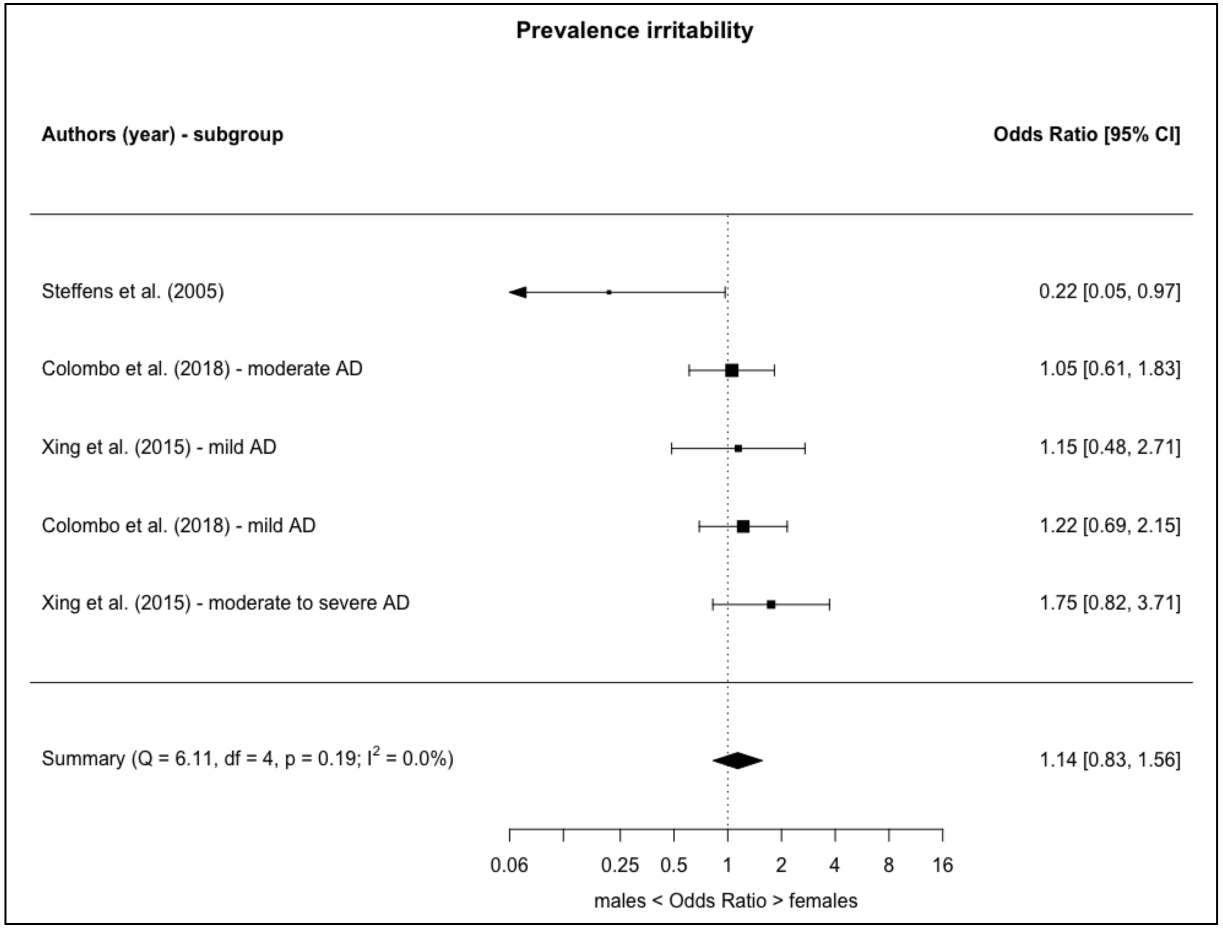

I) Forest plot prevalence sleep disturbances

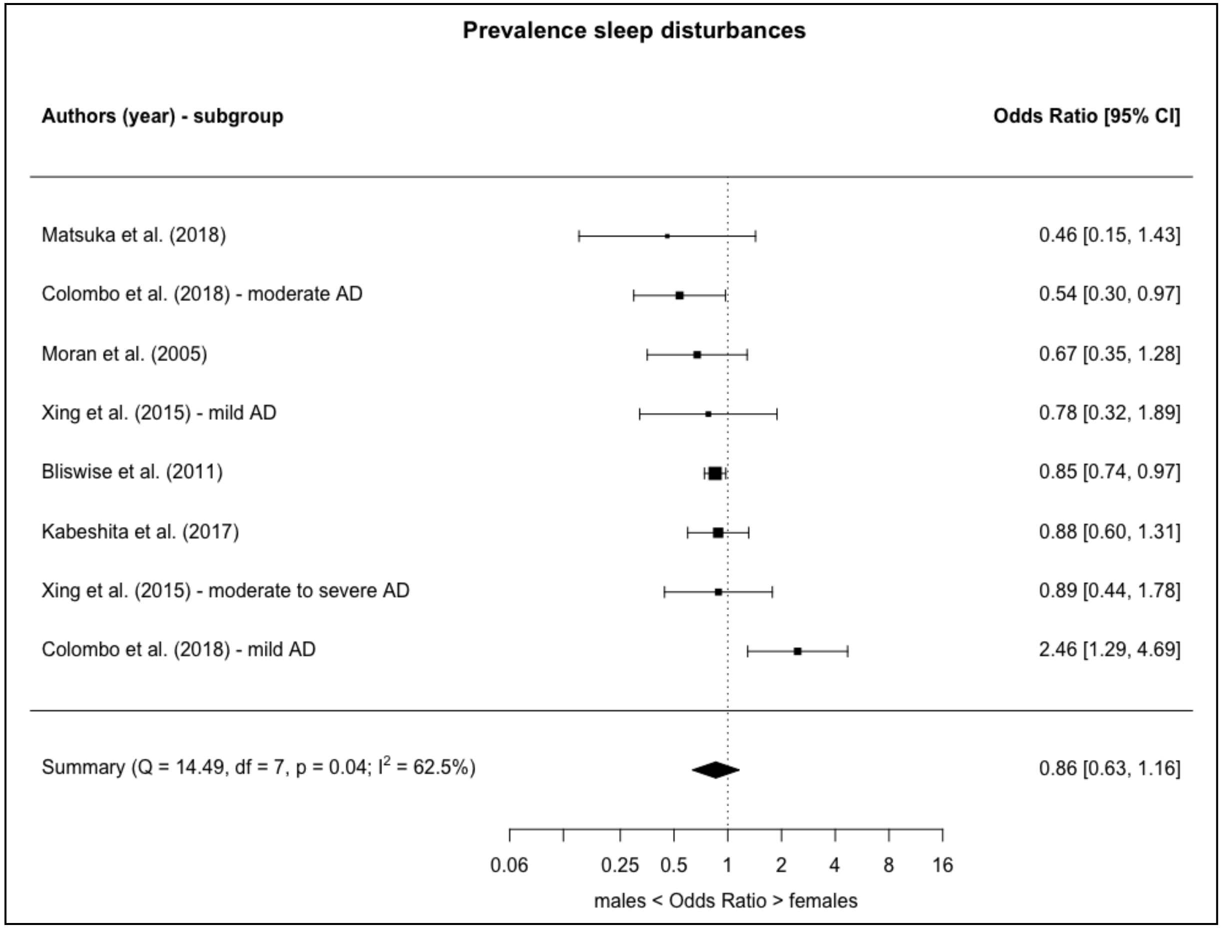

eFigure 2. Forest plots meta-analyses severity specific NPS

A) Forest plot severity agitation

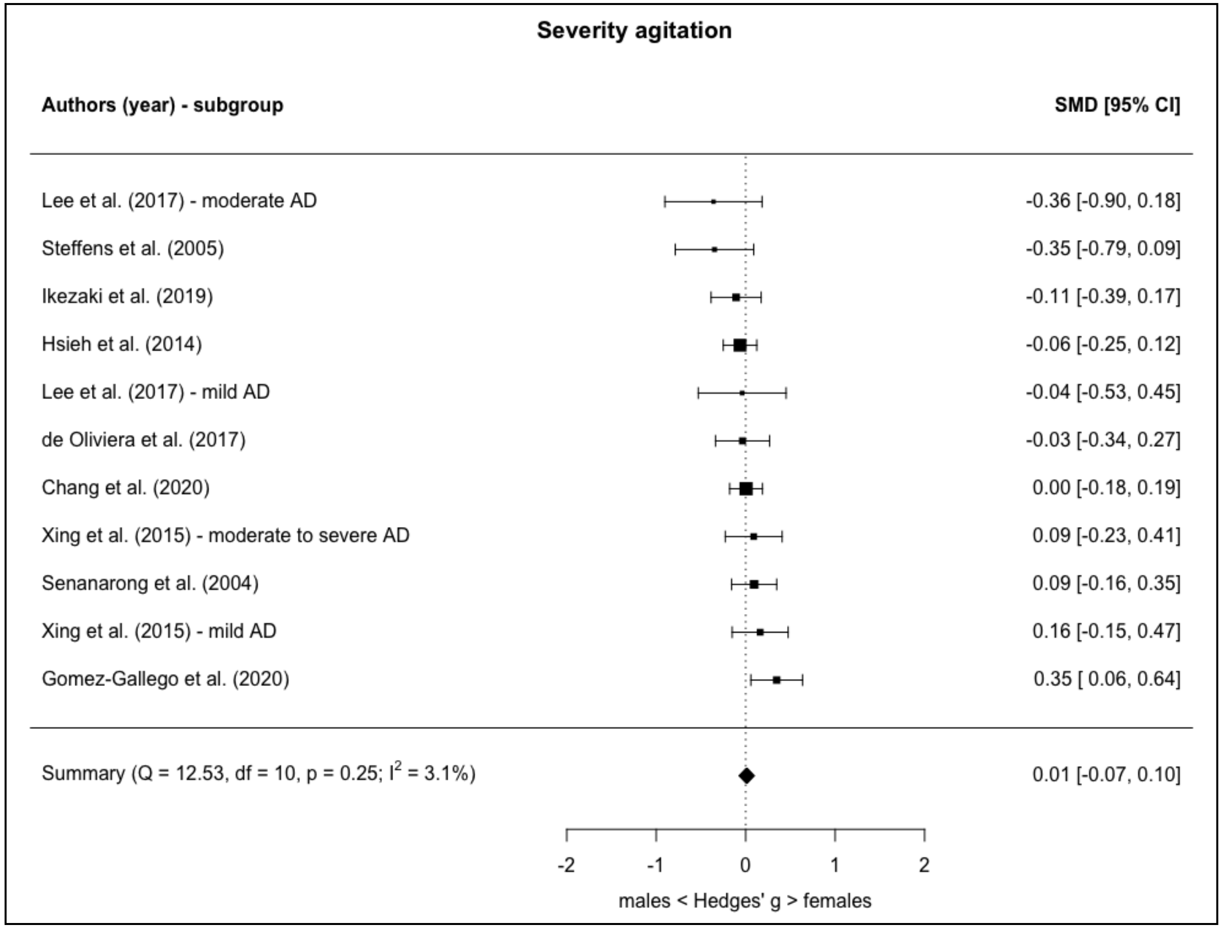

B) Forest plot severity anxiety

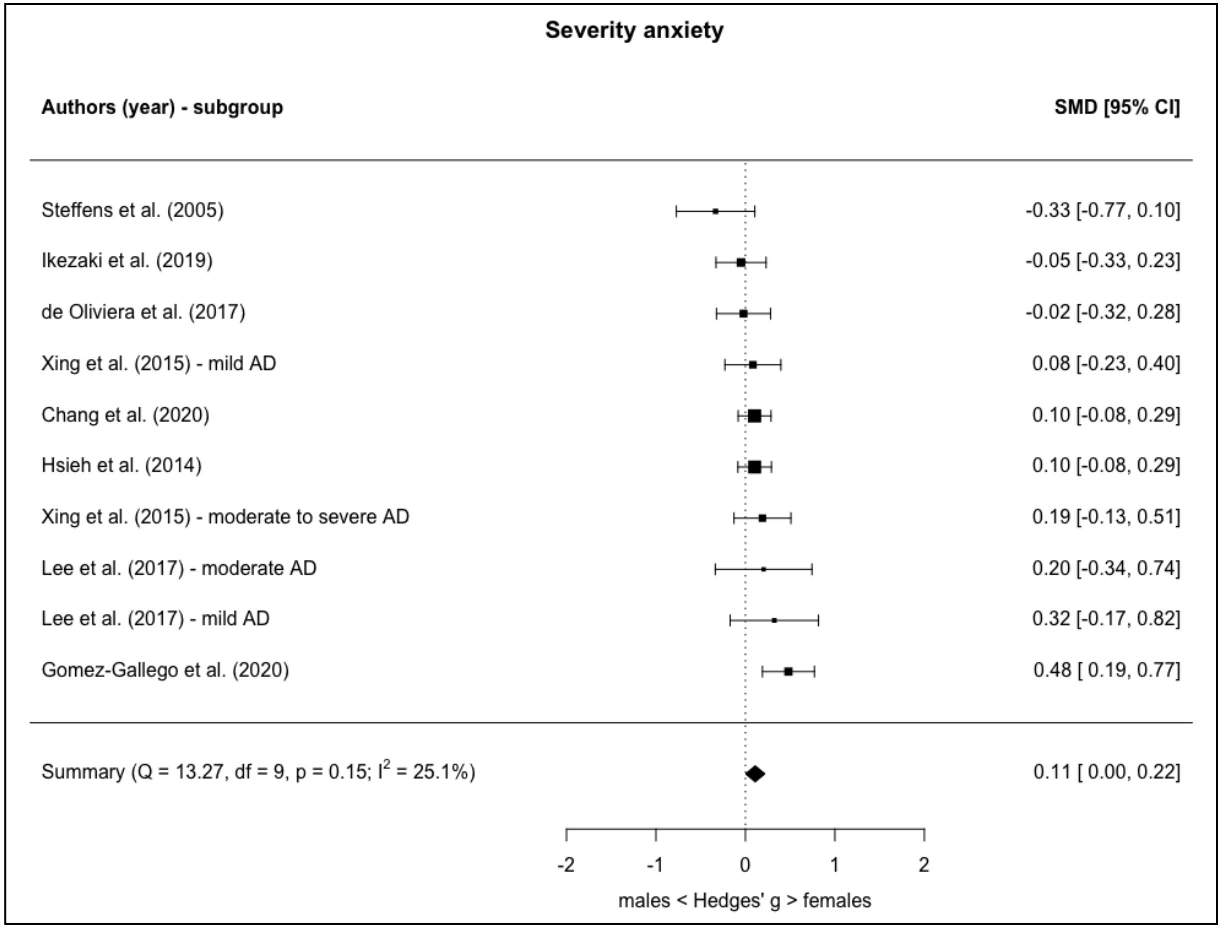

C) Forest plot severity disinhibition

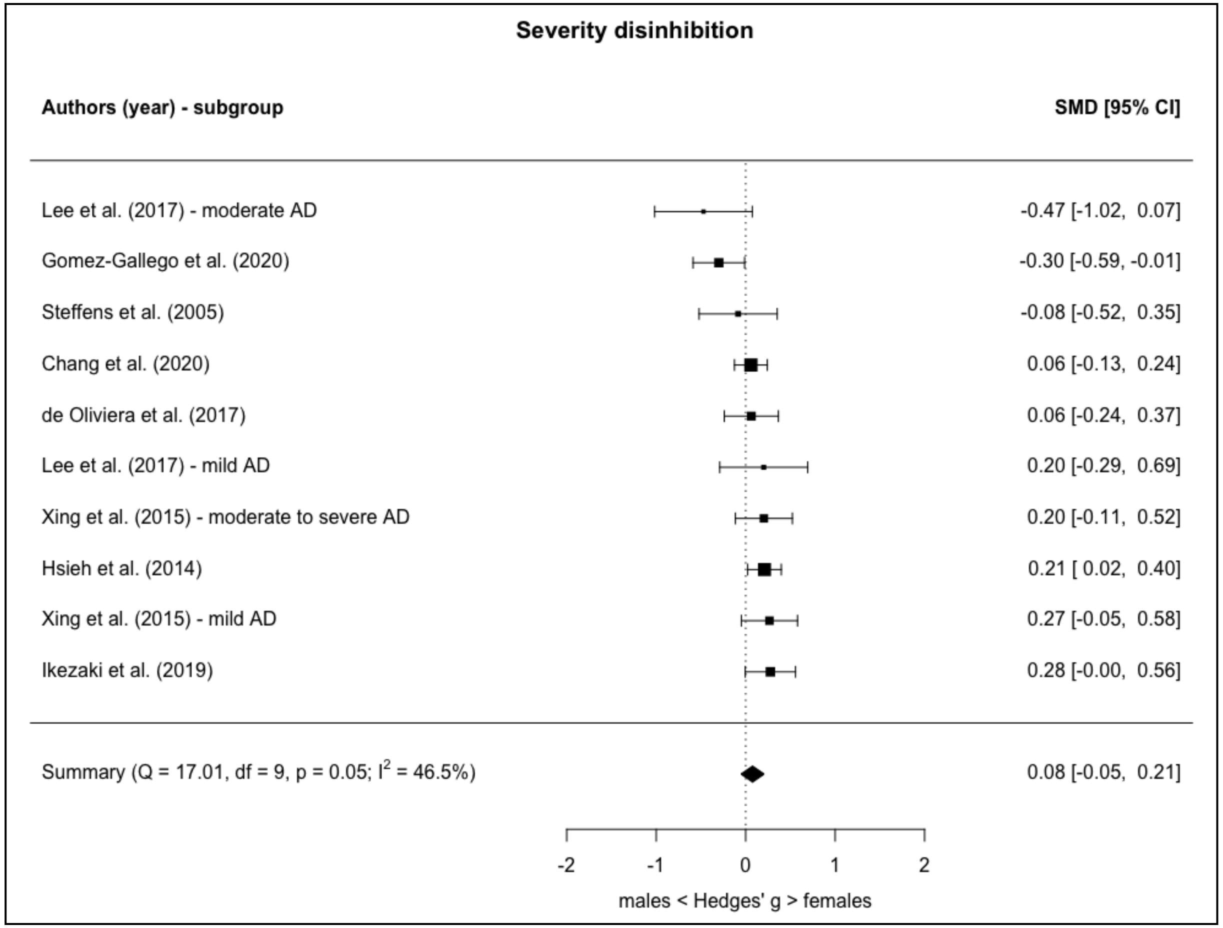

D) Forest plot severity eating behavior

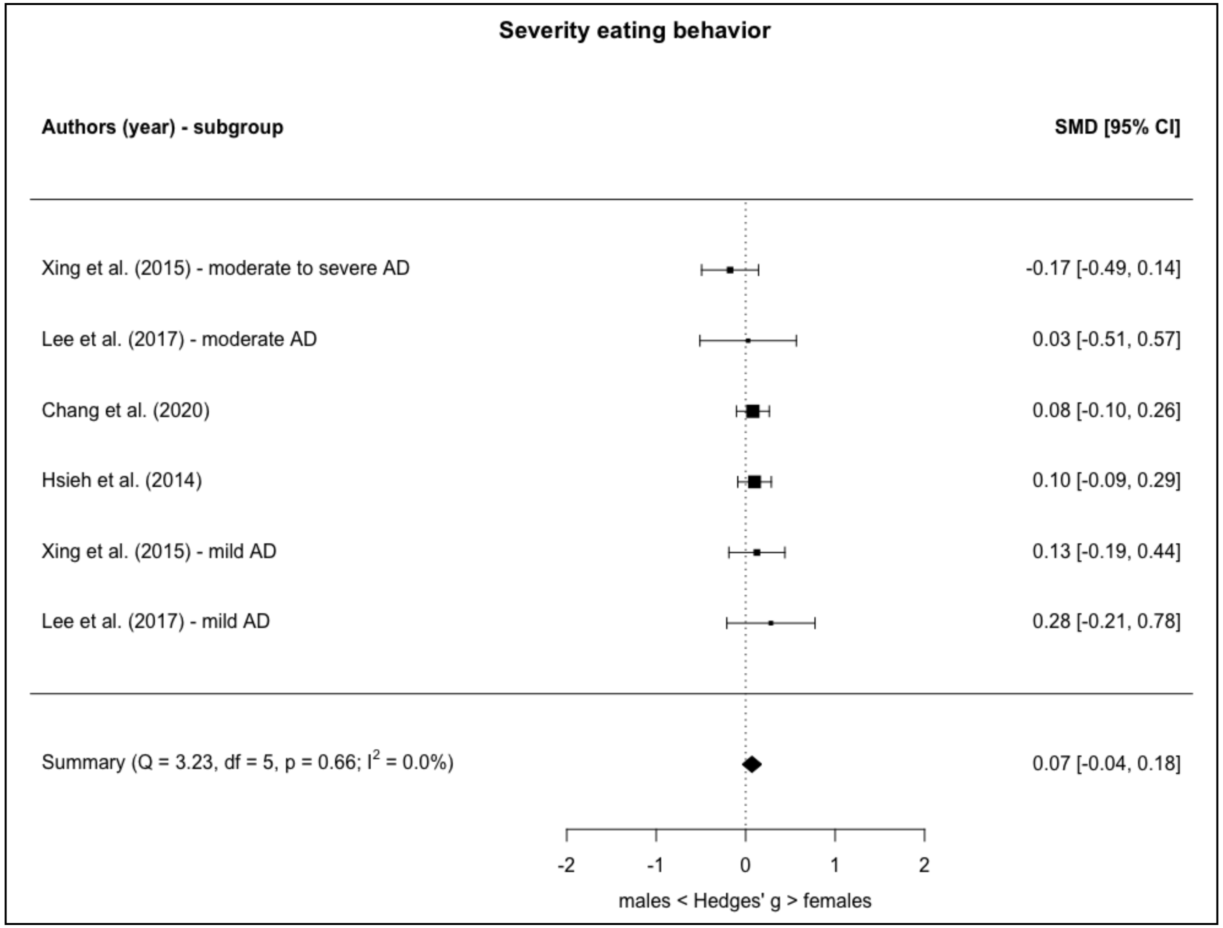

E) Forest plot severity euphoria

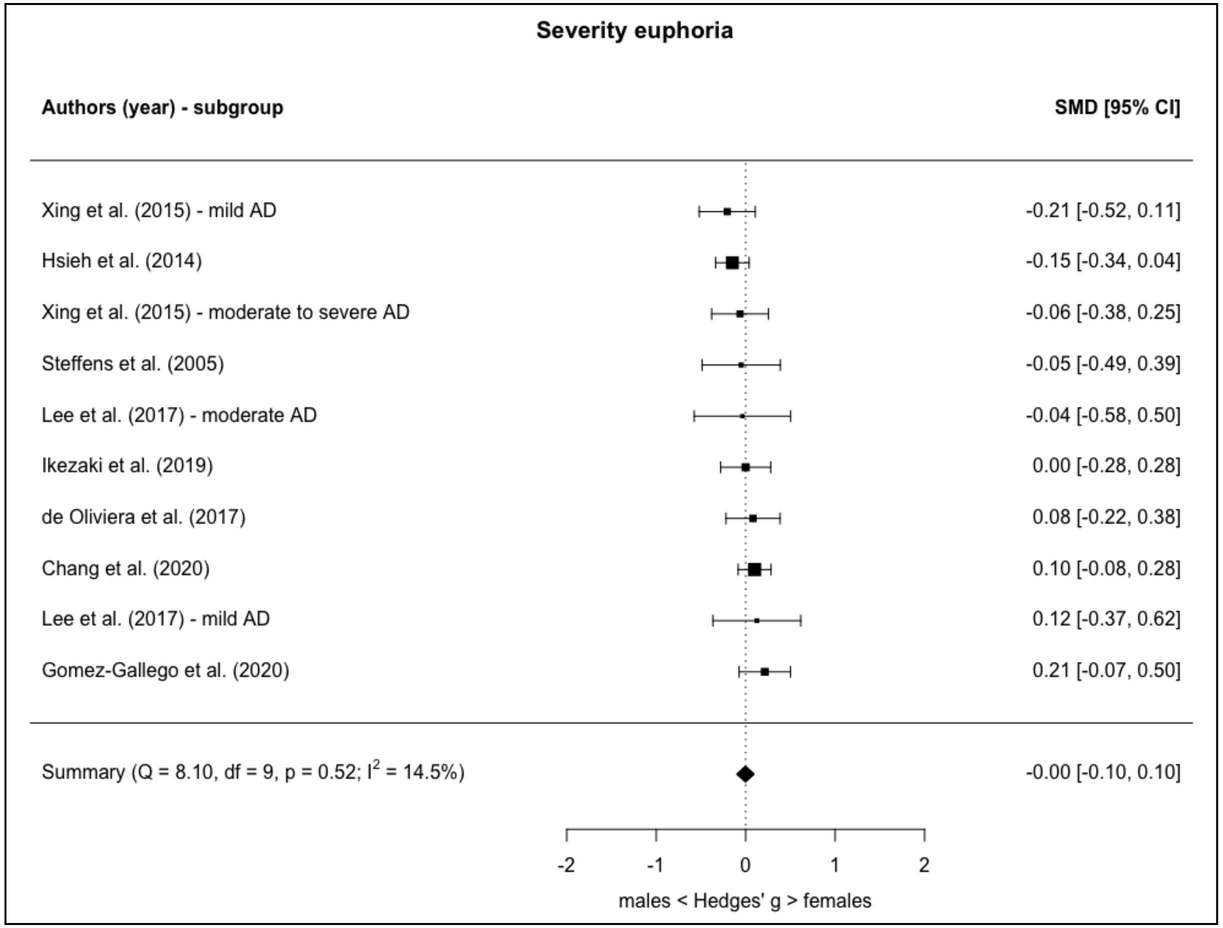

F) Forest plot severity hallucinations

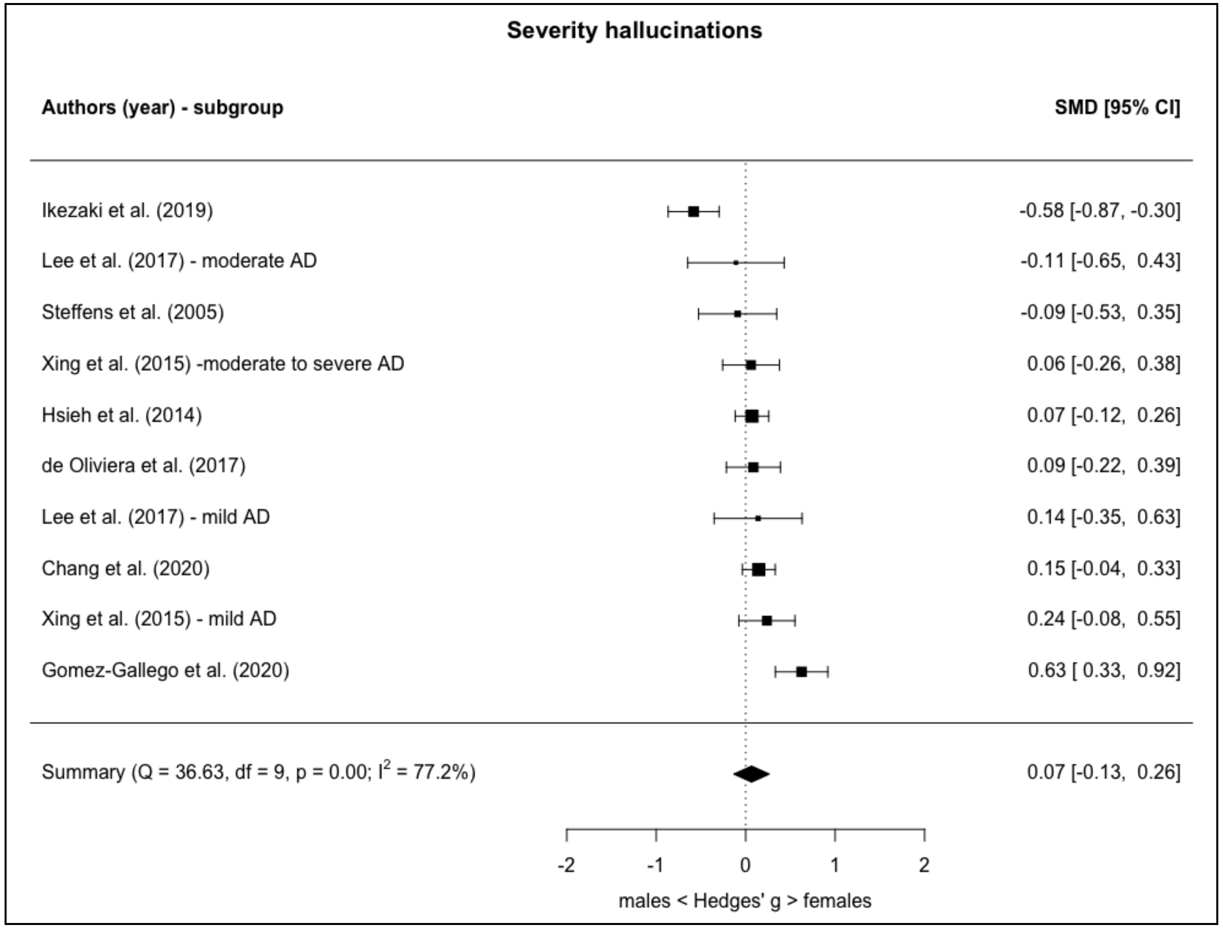

G) Forest plot severity irritability

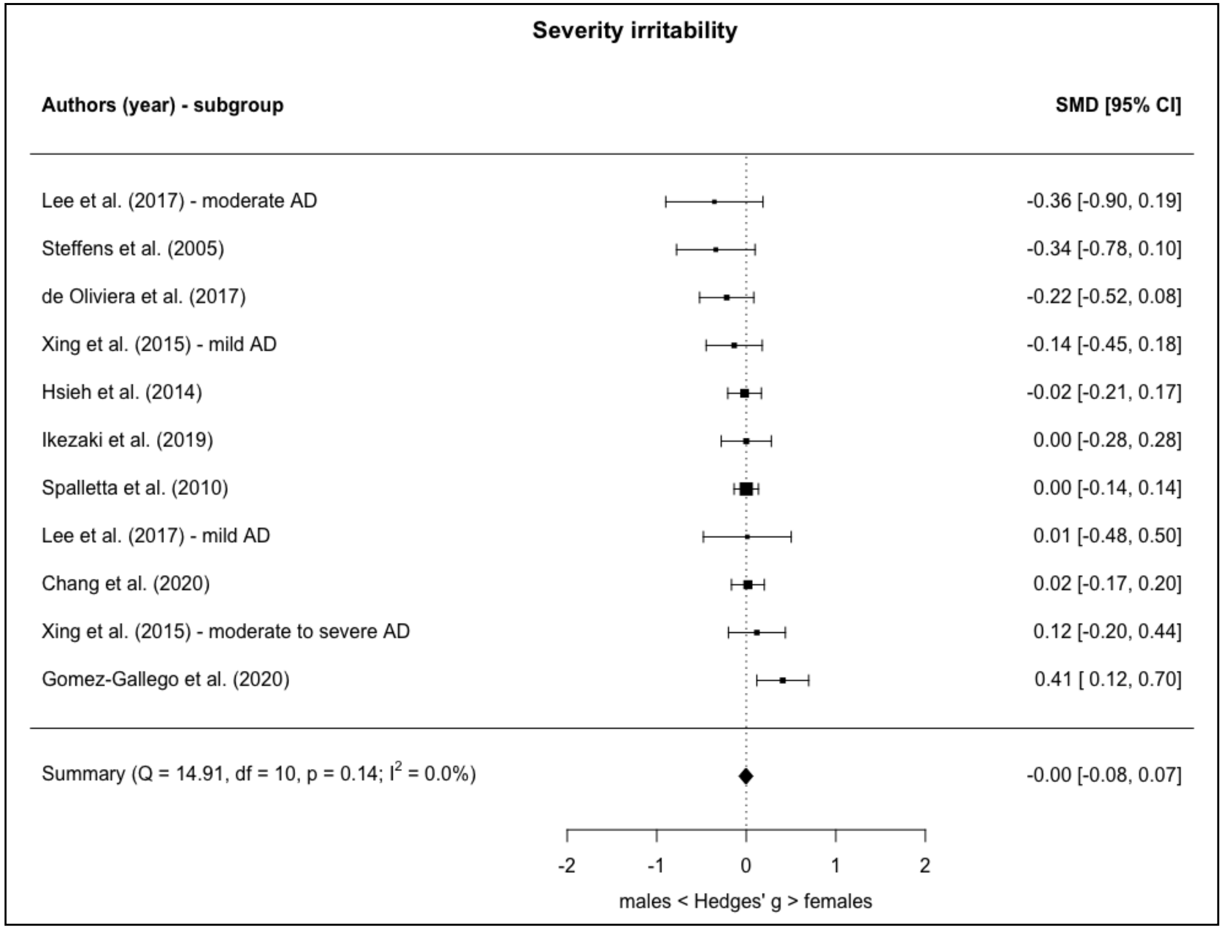

H) Forest plot severity sleep disturbances

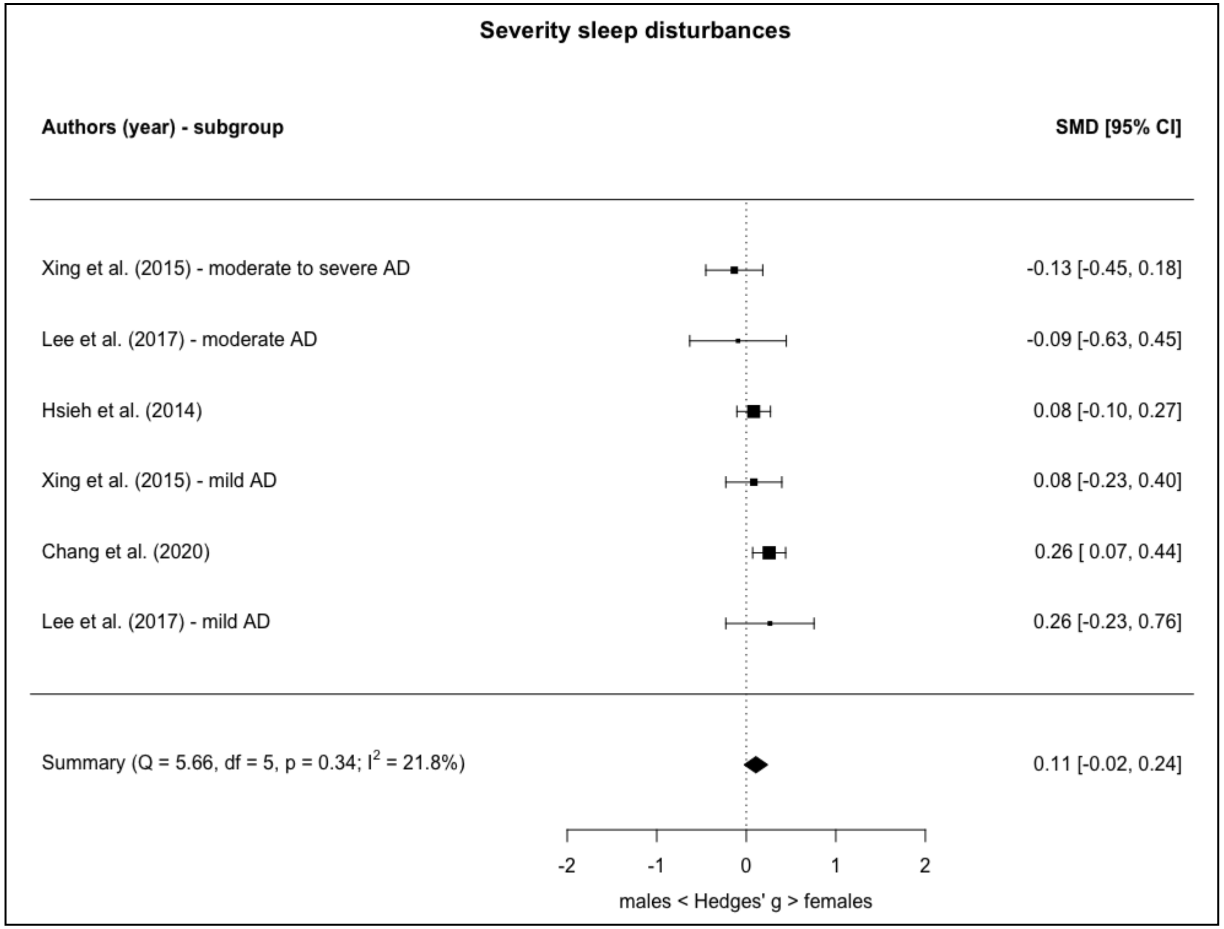

eFigure 3. Funnel plots of meta-analyses specific NPS prevalence

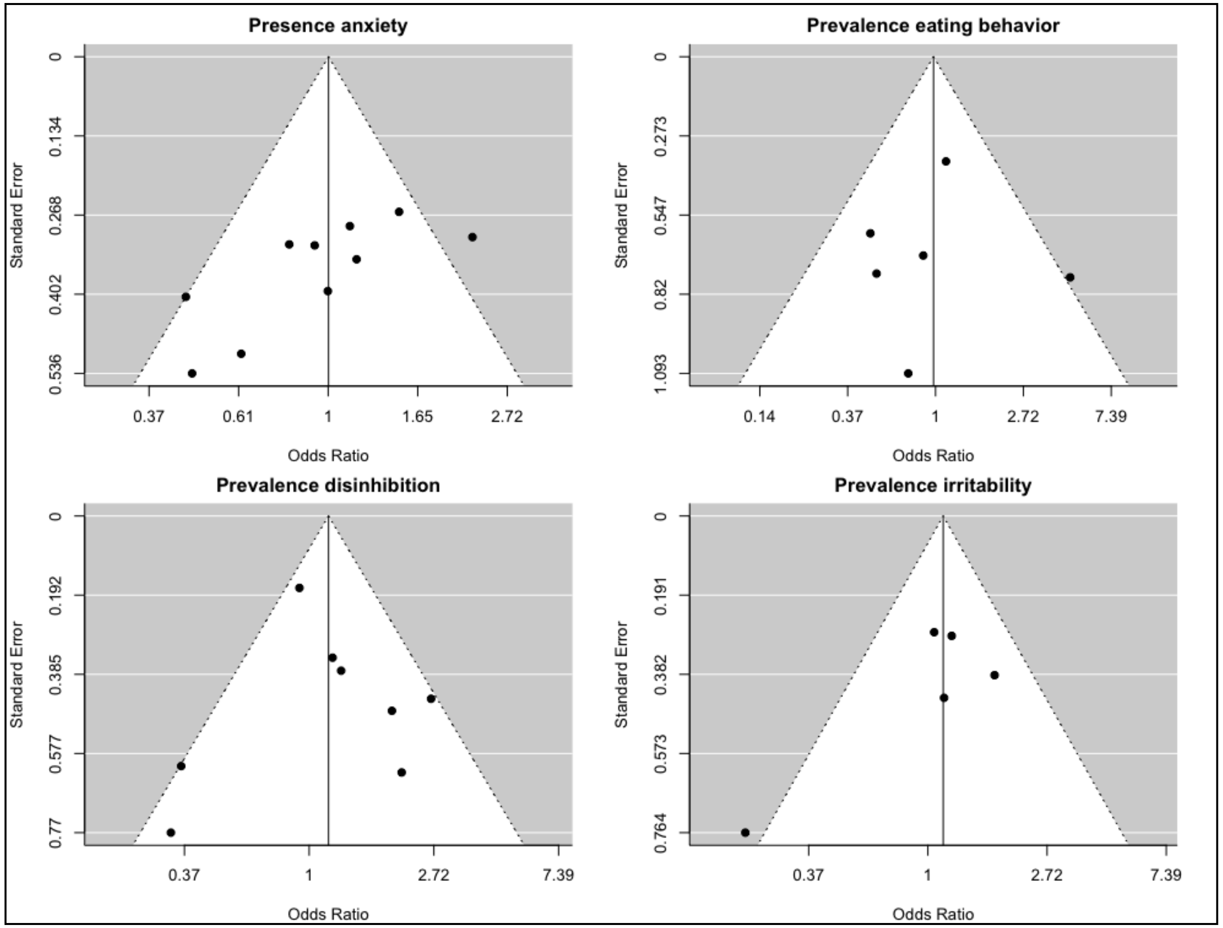

eFigure 3 (continued). Funnel plots of meta-analyses specific NPS prevalence

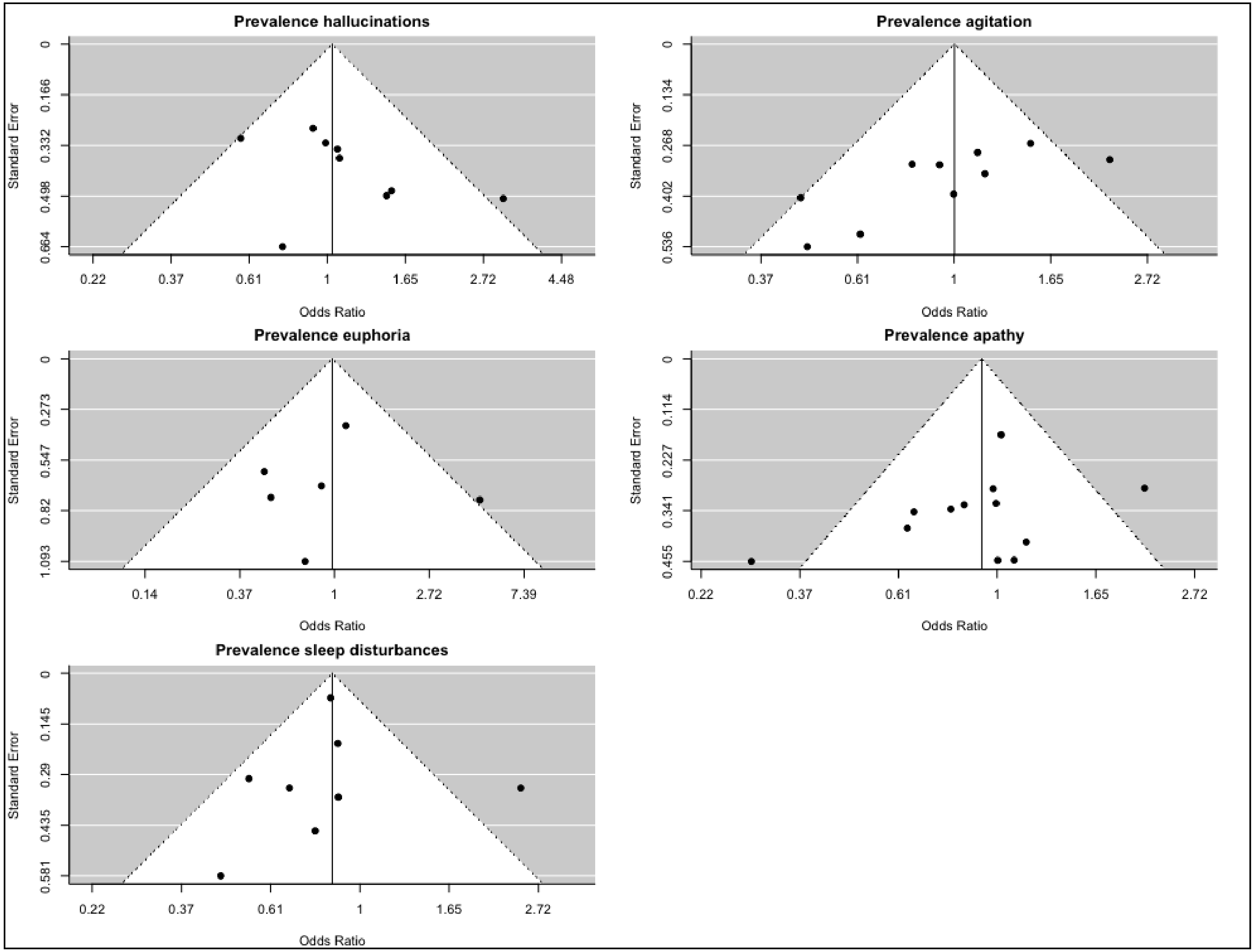

eFigure 4. Funnel plots of meta-analyses specific NPS severity

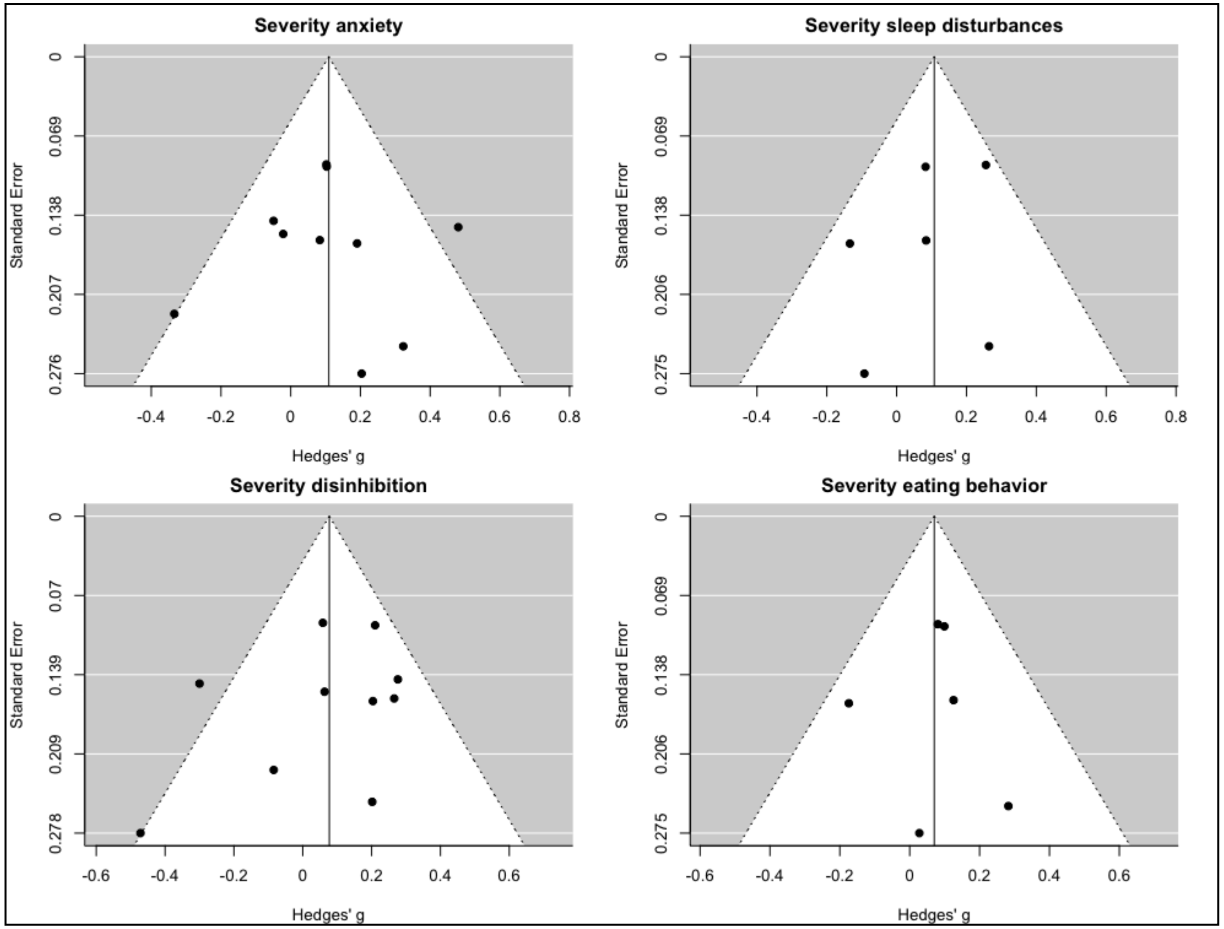

eFigure 4 (contined). Funnel plots of meta-analyses specific NPS severity

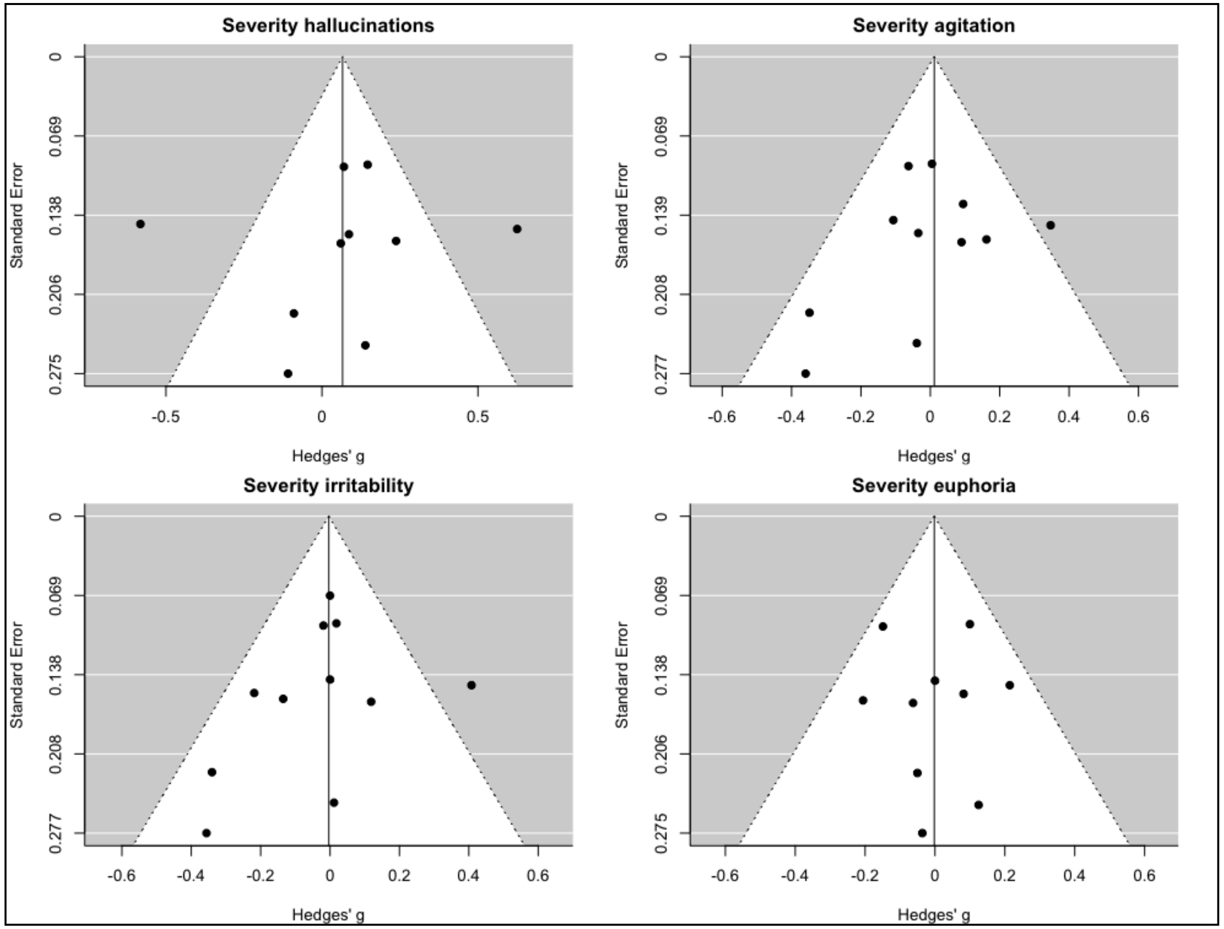

eReferences

1. Alberici A, Bocchio L, Geroldi C, et al. Serum leptin levels are higher in females affected by frontotemporal lobar degeneration than Alzheimer's disease. Journal of Neurology, Neurosurgery and Psychiatry 2008;79:712-715.
2. Álvarez A, Aleixandre M, Linares C, Masliah E, Moessler H. Apathy and APOE4 are associated with reduced BDNF levels in Alzheimer's disease. Journal of Alzheimer's Disease 2014;42:1347-1355.
3. Benoit M, Andrieu S, Lechowski L, Gillette-Guyonnet S, Robert PH, Vellas B. Apathy and depression in Alzheimer's disease are associated with functional deficit and psychotropic prescription. International Journal of Geriatric Psychiatry: A journal of the psychiatry of late life and allied sciences 2008;23:409-414.
4. Bliwise DL, Mercaldo ND, Avidan AY, Boeve BF, Greer SA, Kukull WA. Sleep disturbance in dementia with Lewy bodies and Alzheimer's disease: a multicenter analysis. Dementia and Geriatric Cognitive Disorders 2011;31:239-246.
5. Burns A, Jacoby R, Levy R. Psychiatric phenomena in Alzheimer's disease. III: Disorders of mood. The British Journal of Psychiatry 1990;157:81-86.
6. Burns A, Jacoby R, Levy R. Psychiatric phenomena in Alzheimer's disease. IV: Disorders of behaviour. The British journal of psychiatry 1990;157:86-94.
7. Chang K-H, Huang C-C, Chen C-M, Wu H-C, Kuo H-C. Differences in Clinical Presentation of Behavioral and Psychological Symptoms of Dementia in Alzheimer's Disease According to Sex and Education Level. Journal of Alzheimer's Disease 2020:1-9.
8. Charernboon T, Phanasathit M. Prevalence of neuropsychiatric symptoms in Alzheimer's disease: a cross-sectional descriptive study in Thailand. Journal of the Medical Association of Thailand= Chotmaihet Thangphaet 2014;97:560-565.
9. Chiu P-Y, Steffens D, Chen P-K, Hsu Y-C, Huang H-T, Lai T-J. Depression in Taiwanese patients with Alzheimer's disease determined by the National Institutes of Mental Health Provisional Criteria. International Psychogeriatrics 2012;24:1299.
10. Colombo D, Caltagirone C, Padovani A, et al. Gender differences in neuropsychiatric symptoms in mild to moderate alzheimer's disease patients undergoing switch of cholinesterase inhibitors: a post hoc analysis of the EVOLUTION study. Journal of Women's Health 2018;27:1368-1377.
11. Connors MH, Ames D, Woodward M, Brodaty H. Psychosis and clinical outcomes in Alzheimer disease: a longitudinal study. The American Journal of Geriatric Psychiatry 2018;26:304-313.
12. Del Prete M, Spaccavento S, Craca A, Fiore P, Angelelli P. Neuropsychiatric symptoms and the APOE genotype in Alzheimer's disease. Neurological Sciences 2009;30:367-373.
13. Delano-Wood L, Houston WS, Emond JA, et al. APOE genotype predicts depression in women with Alzheimer's disease: a retrospective study. International Journal of Geriatric Psychiatry 2008;23:632-636.
14. Derouesné C, Guigot J, Chermat V, Winchester N, Lacomblez L. Sexual behavioral changes in Alzheimer disease. Alzheimer Disease and Associated Disorders 1996;10:86-92.
15. Eustace A, Kidd N, Greene E, et al. Verbal aggression in Alzheimer's disease. Clinical, functional and neuropsychological correlates. International Journal of Geriatric Psychiatry 2001;16:858-861.
16. Finger E, Zhang J, Dickerson B, Bureau Y, Masellis M. Disinhibition in Alzheimer's disease is associated with reduced right frontal pole cortical thickness. Journal of Alzheimer's Disease 2017;60:1161-1170.
17. Gomez-Gallego M, Gallego-Mellado M, Gómez-Gallego JC, Gómez-García JG. Sex Moderates the Relationship of Stress and Perceived Social Support With Neuropsychological Symptoms Among Community-dwelling Persons With Alzheimer Disease in Spain. Alzheimer Disease and Associated Disorders 2020.
18. Gormley N, Rizwan MR, Lovestone S. Clinical predictors of aggressive behaviour in Alzheimer's disease. International Journal of Geriatric Psychiatry 1998;13:109-115.
19. Gormley N, Rizwan MR. Prevalence and clinical correlates of psychotic symptoms in Alzheimer's disease. International Journal of Geriatric Psychiatry 1998;13:410-414.
20. Hall JR, Wiechmann AR, Johnson LA, et al. Total cholesterol and neuropsychiatric symptoms in Alzheimer's disease: The impact of total cholesterol level and gender. Dementia and Geriatric Cognitive Disorders 2014;38:300-309.
21. Ikezaki H, Hashimoto M, Ishikawa T, et al. Relationship between executive dysfunction and neuropsychiatric symptoms and impaired instrumental activities of daily living among patients with very mild Alzheimer's disease. International Journal of Geriatric Psychiatry 2020;35:877-887.
22. Hsieh S-W, Chen C-H, Huang L-C, Chang Y-H, Yang Y-H. Gender differences in presentation of behavioral and psychological symptoms in Alzheimer's disease in Taiwan. Aging & Mental Health 2020;24:1342-1347.
23. Kabeshita Y, Adachi H, Matsushita M, et al. Sleep disturbances are key symptoms of very early stage Alzheimer disease with behavioral and psychological symptoms: a Japan multi-center cross-sectional study (J-BIRD). International Journal of Geriatric Psychiatry 2017;32:222-230.

24. Kaiser NC, Liang L-J, Melrose RJ, Wilkins SS, Sultzer DL, Mendez MF. Differences in anxiety among patients with early-versus late-onset Alzheimer’s disease. The Journal of neuropsychiatry and clinical neurosciences 2014;26:73-80.
25. Karttunen K, Karppi P, Hiltunen A, et al. Neuropsychiatric symptoms and quality of life in patients with very mild and mild Alzheimer's disease. International Journal of Geriatric Psychiatry 2011;26:473-482.
26. Kaufer DI, Cummings JL, Christine D, et al. Assessing the impact of neuropsychiatric symptoms in Alzheimer's disease: the Neuropsychiatric Inventory Caregiver Distress Scale. Journal of the American Geriatrics Society 1998;46:210-215.
27. Kwak YT, Yang Y, Kwak SG, Koo MS. Delusions of Korean patients with Alzheimer's disease: Study of drug-naïve patients. Geriatrics & gerontology international 2013;13:307-313.
28. Landes AM, Sperry SD, Strauss ME. Prevalence of apathy, dysphoria, and depression in relation to dementia severity in Alzheimer’s disease. The Journal of neuropsychiatry and clinical neurosciences 2005;17:342-349.
29. Lee J, Lee KJ, Kim H. Gender differences in behavioral and psychological symptoms of patients with Alzheimer's disease. Asian Journal of Psychiatry 2017;26:124-128.
30. Leroi I, Voulgari A, Breitner JC, Lyketsos CG. The epidemiology of psychosis in dementia. American Journal of Geriatric Psychiatry 2003;11:83-91.
31. Levy ML, Cummings JL, Fairbanks LA, Bravi D, Calvani M, Carta A. Longitudinal assessment of symptoms of depression, agitation, and psychosis in 181 patients with Alzheimer's disease. The American journal of psychiatry 1996;153:1438-1443.
32. Linszen MMJ, Lemstra AW, Dauwan M, Brouwer RM, Scheltens P, Sommer IEC. Understanding hallucinations in probable Alzheimer's disease: Very low prevalence rates in a tertiary memory clinic. Alzheimer's & dementia (Amsterdam, Netherlands) 2018;10:358-362.
33. Liu CY, Fuh JL, Teng EL, et al. Depressive disorders in Chinese patients with Alzheimer's disease. Acta Psychiatrica Scandinavica 1999;100:451-455.
34. Matsuoka T, Imai A, Fujimoto H, et al. Neural correlates of sleep disturbance in Alzheimer’s disease: Role of the precuneus in sleep disturbance. Journal of Alzheimer's Disease 2018;63:957-964.
35. Migliorelli R, Teson A, Sabe L, Petracchi M, Leiguarda R, Starkstein SE. Prevalence and correlates of dysthymia and major depression among patients with Alzheimer's disease. The American journal of psychiatry 1995.
36. Moran M, Lynch CA, Walsh C, Coen R, Coakley D, Lawlor BA. Sleep disturbance in mild to moderate Alzheimer's disease. Sleep Medicine 2005;6:347-352.
37. Moran EK, Becker JA, Satlin A, Lyoo IK, Fischman AJ, Johnson KA. Psychosis of Alzheimer's disease: Gender differences in regional perfusion. Neurobiology of Aging 2008;29:1218-1225.
38. Müller-Thomsen T, Arlt S, Ganzer S, et al. Depression in Alzheimer’s disease might be associated with apolipoprotein E ε4 allele frequency in women but not in men. Dementia and Geriatric Cognitive Disorders 2002;14:59-63.
39. Mulin E, Leone E, Dujardin K, et al. Diagnostic criteria for apathy in clinical practice. International Journal of Geriatric Psychiatry 2011;26:158-165.
40. Oliveira FF, Chen ES, Smith MC, Bertolucci PH. Associations of cerebrovascular metabolism genotypes with neuropsychiatric symptoms and age at onset of Alzheimer's disease dementia. Braz J Psychiatry 2017;39:95-103.
41. Ott BR, Tate CA, Gordon NM, Heindel WC. Gender differences in the behavioral manifestations of Alzheimer's disease. Journal of the American Geriatrics Society 1996;44:583-587.
42. Porta-Etessam J, Tobaruela-González JL, Rabes-Berendes C. Depression in patients with moderate Alzheimer disease: a prospective observational cohort study. Alzheimer Disease and Associated Disorders 2011;25:317-325.
43. Pusswald G, Lehrner J, Hagmann M, et al. Gender-Specific Differences in Cognitive Profiles of Patients with Alzheimer's Disease: Results of the Prospective Dementia Registry Austria (PRODEM-Austria). Journal of Alzheimer's disease : JAD 2015;46:631-637.
44. Rozzini L, Chilovi BV, Bertoletti E, Trabucchi M, Padovani A. Acetylcholinesterase inhibitors and depressive symptoms in patients with mild to moderate Alzheimer’s disease. Aging Clinical and Experimental Research 2007;19:220-223.
45. Shi Q, Zhou F, Mei J, Yang H, Li H. The Effect of Type 2 Diabetes Mellitus on Neuropsychological Symptoms in Chinese Early Alzheimer’s Disease Population. Neuropsychiatric Disease and Treatment 2020;16:829.
46. Senanarong V, Cummings JL, Fairbanks L, et al. Agitation in Alzheimer’s disease is a manifestation of frontal lobe dysfunction. Dementia and Geriatric Cognitive Disorders 2004;17:14-20.
47. Sinforiani E, Citterio A, Zucchella C, et al. Impact of gender differences on the outcome of Alzheimer’s disease. Dementia and Geriatric Cognitive Disorders 2010;30:147-154.

48. Slifer MA, Martin ER, Gilbert JR, Haines JL, Pericak-Vance MA. Resolving the relationship between ApolipoproteinE and depression. Neuroscience Letters 2009;455:116-119.
49. Spalletta G, Musicco M, Padovani A, et al. Neuropsychiatric symptoms and syndromes in a large cohort of newly diagnosed, untreated patients with Alzheimer disease. The American Journal of Geriatric Psychiatry 2010;18:1026-1035.
50. Steffens DC, Maytan M, Helms MJ, Plassman BL. Prevalence and clinical correlates of neuropsychiatric symptoms in dementia. American Journal of Alzheimer's Disease & Other Dementias® 2005;20:367-373.
51. Suma S, Watanabe Y, Hirano H, et al. Factors affecting the appetites of persons with Alzheimer's disease and mild cognitive impairment. Geriatrics & gerontology international 2018;18:1236-1243.
52. Toyota Y, Ikeda M, Shinagawa S, et al. Comparison of behavioral and psychological symptoms in early-onset and late-onset Alzheimer's disease. International Journal of Geriatric Psychiatry: A journal of the psychiatry of late life and allied sciences 2007;22:896-901.
53. Treiber KA, Lyketsos CG, Corcoran C, et al. Vascular factors and risk for neuropsychiatric symptoms in Alzheimer’s disease: the Cache County Study. International psychogeriatrics/IPA 2008;20:538.
54. Troisi A, Pasini A, Gori G, Sorbi T, Baroni A, Ciani N. Clinical predictors of somatic and psychological symptoms of depression in Alzheimer's disease. International Journal of Geriatric Psychiatry 1996;11:23-27.
55. Tunnard C, Whitehead D, Hurt C, et al. Apathy and cortical atrophy in Alzheimer's disease. International Journal of Geriatric Psychiatry 2011;26:741-748.
56. Usman S, Chaudhary HR, Asif A, Yahya MI. Severity and risk factors of depression in Alzheimer’s disease. Journal of the College of Physicians and Surgeons--Pakistan 2010;20:327-330.
57. Vilalta-Franch J, López-Pousa S, Calvó-Perxas L, Garre-Olmo J. Psychosis of Alzheimer disease: prevalence, incidence, persistence, risk factors, and mortality. The American Journal of Geriatric Psychiatry 2013;21:1135-1143.
58. Xing Y, Tang Y, Jia J. Sex differences in neuropsychiatric symptoms of Alzheimer’s disease: the modifying effect of apolipoprotein E ε4 status. Behavioural Neurology 2015;2015.
59. Yang B, Yu H, Xing M, He R, Liang R, Zhou L. The relationship between cognition and depressive symptoms, and factors modifying this association, in Alzheimer’s disease: A multivariate multilevel model. Archives of Gerontology and Geriatrics 2017;72:25-31.
60. Yang H, Hong W, Chen L, Tao Y, Peng Z, Zhou H. Analysis of risk factors for depression in Alzheimer’s disease patients. International Journal of Neuroscience 2020;130:1136-1141.
61. Zeitzer JM, David R, Friedman L, et al. Phenotyping apathy in individuals with Alzheimer disease using functional principal component analysis. The American Journal of Geriatric Psychiatry 2013;21:391-397.
62. Zhao H, Zhao Z, Huang L, Preter M. Diagnosis and assessment of apathy in Chinese patients with Alzheimer's disease. Journal of Psychosomatic Research 2012;72:405-407.
